# Supplementary material for: Within-host diversity and phased variant analysis reveal structures and recombination of Helicobacter pylori subpopulations in stomach
Source: Gigascience. 2026 Apr 16;15:giag046. doi: 10.1093/gigascience/giag046 (PMC13188224; doi:10.1093/gigascience/giag046)
Supplement: giag046_Supplemental_Files [file giag046_supplemental_files.zip › supplementary figure/supplementary-figure.docx]

**
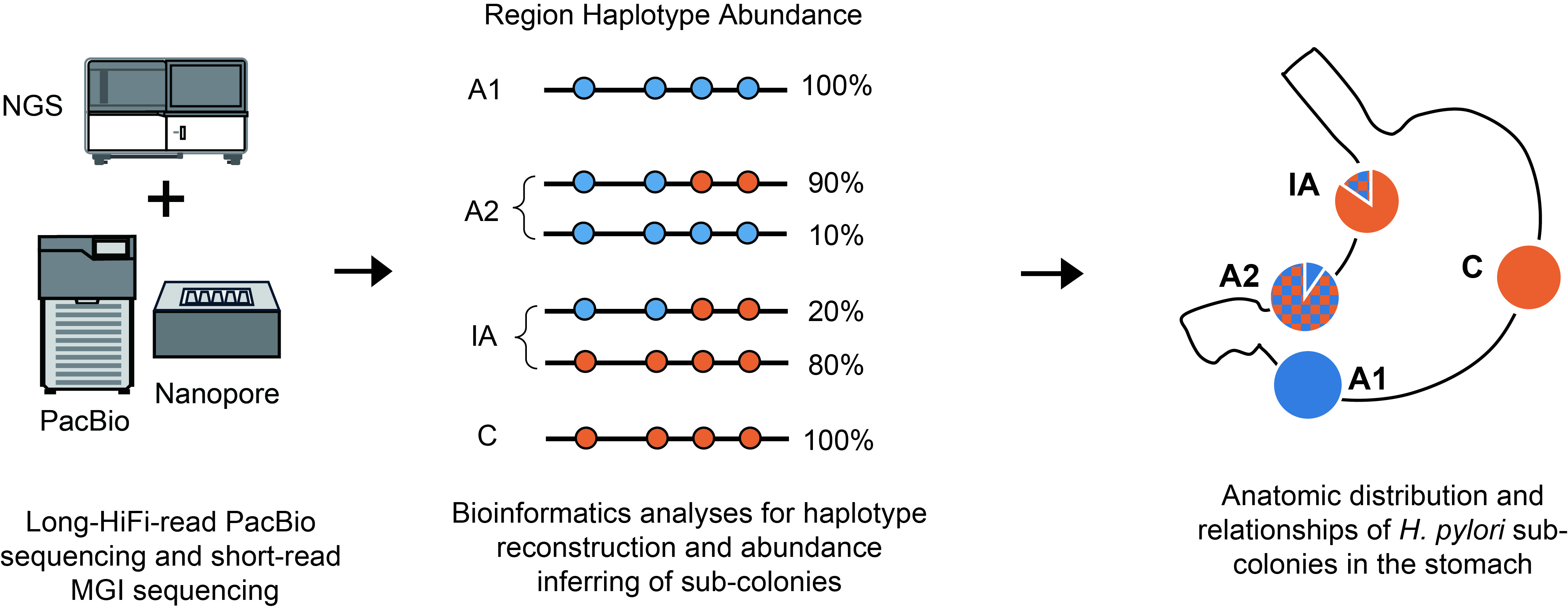
**

**Fig. S1. Workflow for reconstructing within-host *H. pylori* subpopulations across gastric regions.** PacBio HiFi and Nanopore long-read sequencing and short-read MGI sequencing were used to generate whole-genome sequences of *H. pylori* isolates collected from multiple gastric regions (A1, A2, IA, and C). These data were integrated into a bioinformatics pipeline to reconstruct haplotypes and estimate the relative abundance of subpopulations in each region. The inferred within-host subpopulation compositions were then mapped onto a schematic stomach to illustrate their anatomical distribution.


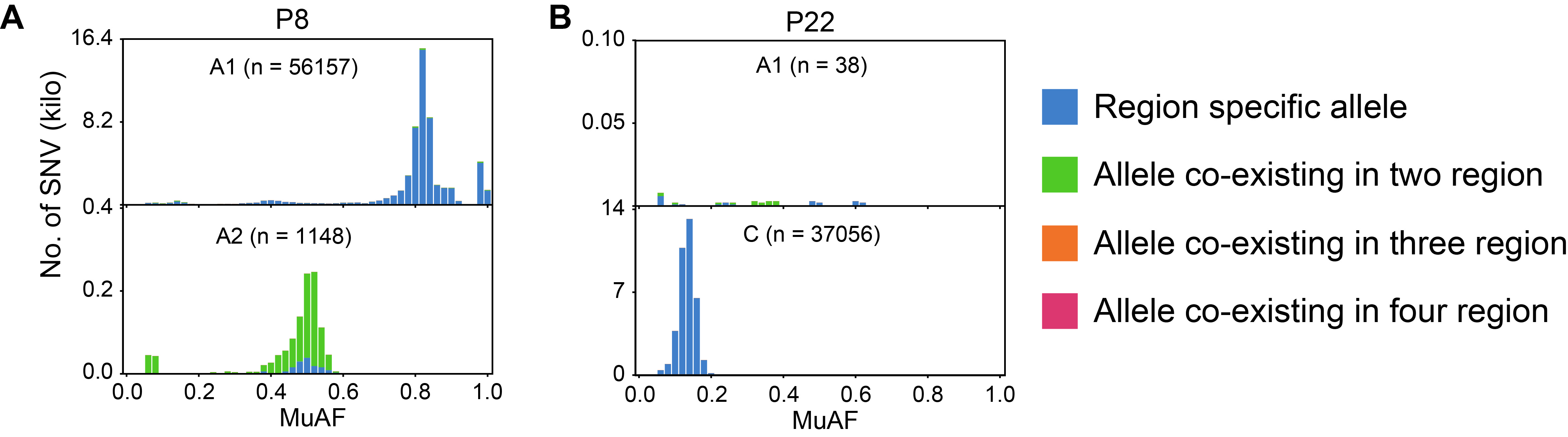


**Fig. S2. MuAF distributions of SNVs in *H. pylori* isolates from P8 and P22 with mixed infection.** (A–B) Histograms show the mutant allele frequency (MuAF) distributions of SNVs in *H. pylori* isolates from patients P8 and P22 with mixed infection. For each gastric region, the total number of SNV sites is shown in parentheses. Bars are colored by regional sharing patterns: region-specific SNVs (blue), SNVs shared between two regions (green), three regions (orange), or all four regions (red).


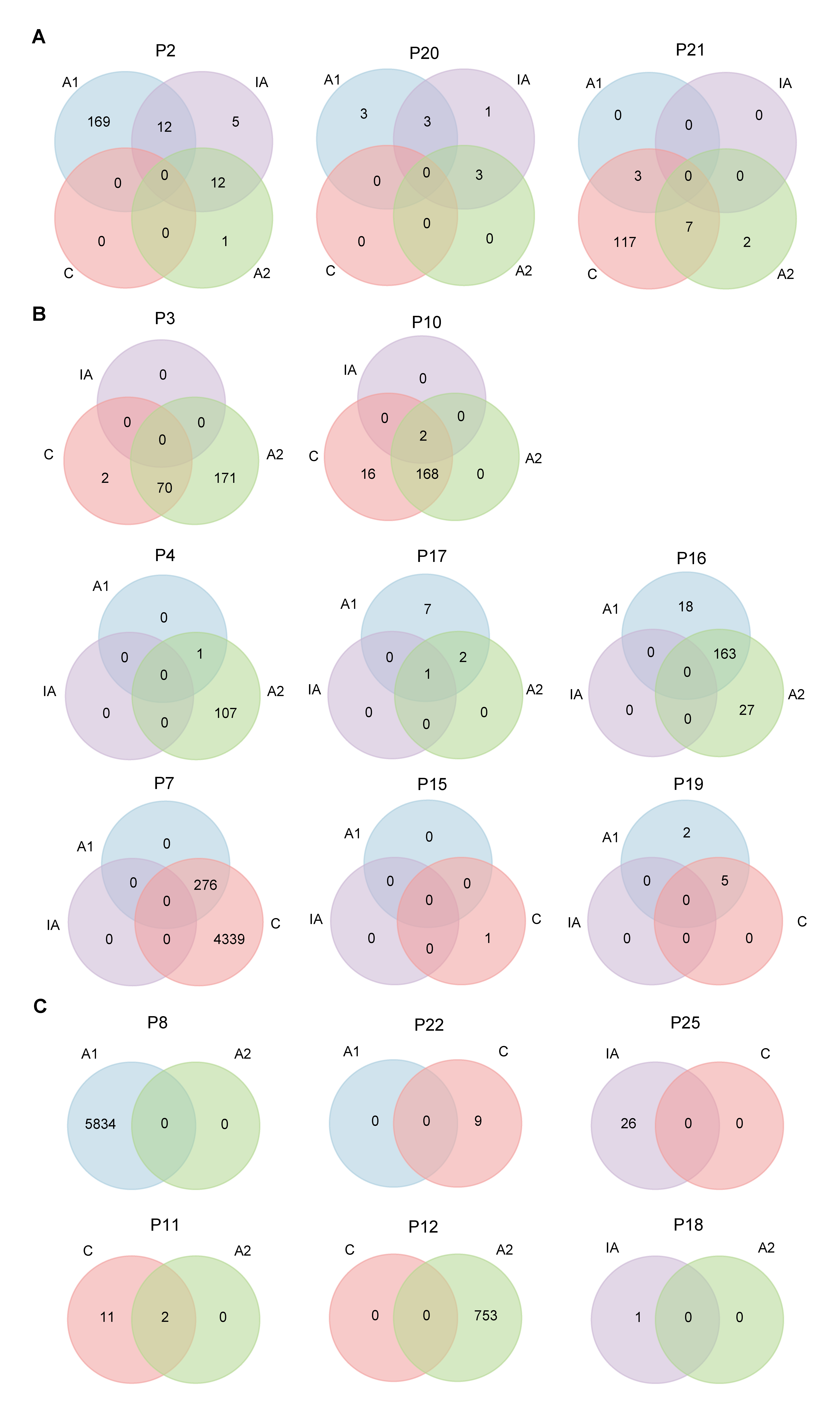


**Fig. S3. Venn diagrams of SNP sharing among gastric regions in H. pylori isolates from individual patients.** Patients are grouped by the number of sampled gastric regions. Each diagram shows the numbers of SNPs unique to, or shared among, the sampled gastric regions within the same patient. Only SNPs with MuAF ≥ 0.95 were included. Gastric regions are labeled as A1, greater curvature of the antrum; A2, lesser curvature of the antrum; IA, incisura angularis; and C, corpus.


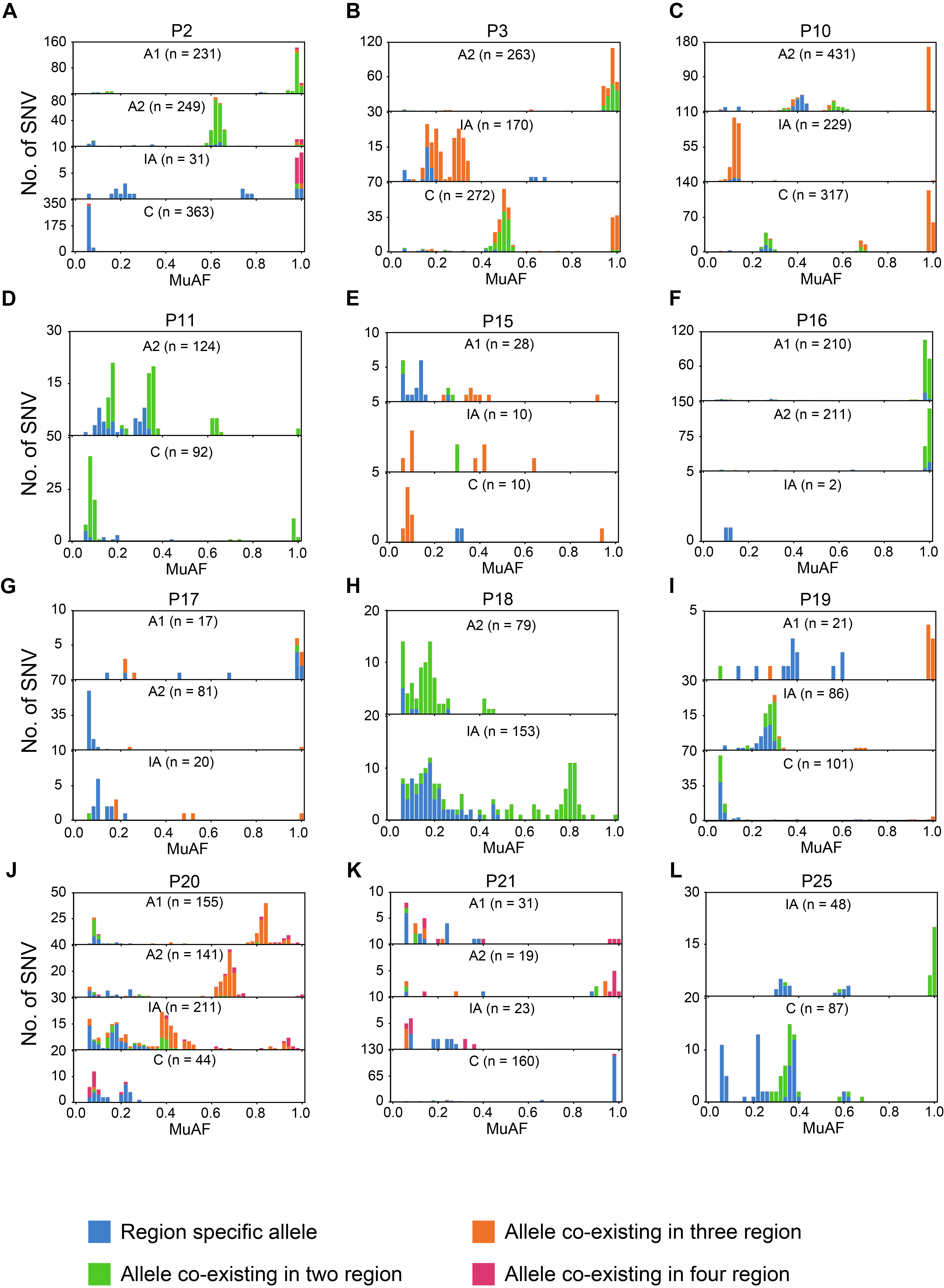


**Fig. S4. MuAF distributions of SNVs in *H. pylori* isolates from patients with lower intra-host variant burdens.** (A–L) Histograms show the MuAF distributions of SNVs in *H. pylori* isolates from 11 patients (P2, P3, P10, P11, P15–P21, and P25), each with <500 SNVs across all sampled gastric regions. For each gastric region, the total number of SNV sites is shown in parentheses. Bars are colored by regional sharing patterns: region-specific SNVs (blue), SNVs shared between two regions (green), three regions (orange), or all four regions (red).


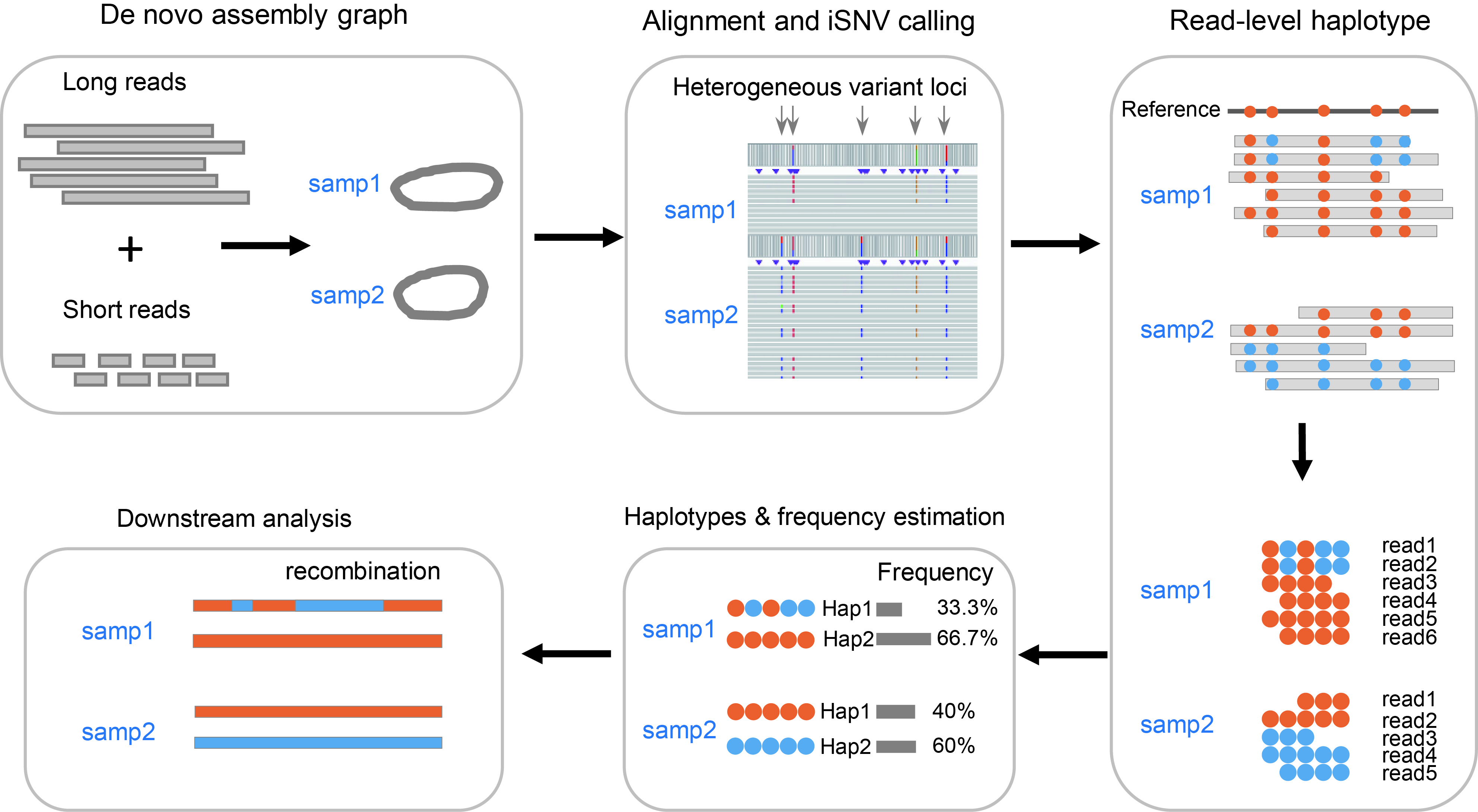


**Fig. S5. Workflow for haplotype phasing and subpopulation inference.** Schematic overview of the phasing pipeline. Long-read and short-read data were combined to perform de novo assembly for each *H. pylori* isolate, with long reads used for contig construction and short reads for polishing, yielding high-quality whole-genome assemblies. Short reads were then mapped back to the corresponding assemblies to identify heterogeneous variant loci (MuAF ≥ 0.05). Long reads spanning at least two variant sites were encoded as allele strings (reference alleles in yellow and variant alleles in blue) to generate read-level haplotypes. These read-level haplotypes were clustered to define local haplotypes, and their relative frequencies were estimated from the numbers of supporting long reads. Finally, phased haplotypes and their frequencies were compared across isolates to infer shared and region-specific recombination tracts and to reconstruct within-host subpopulation structure.


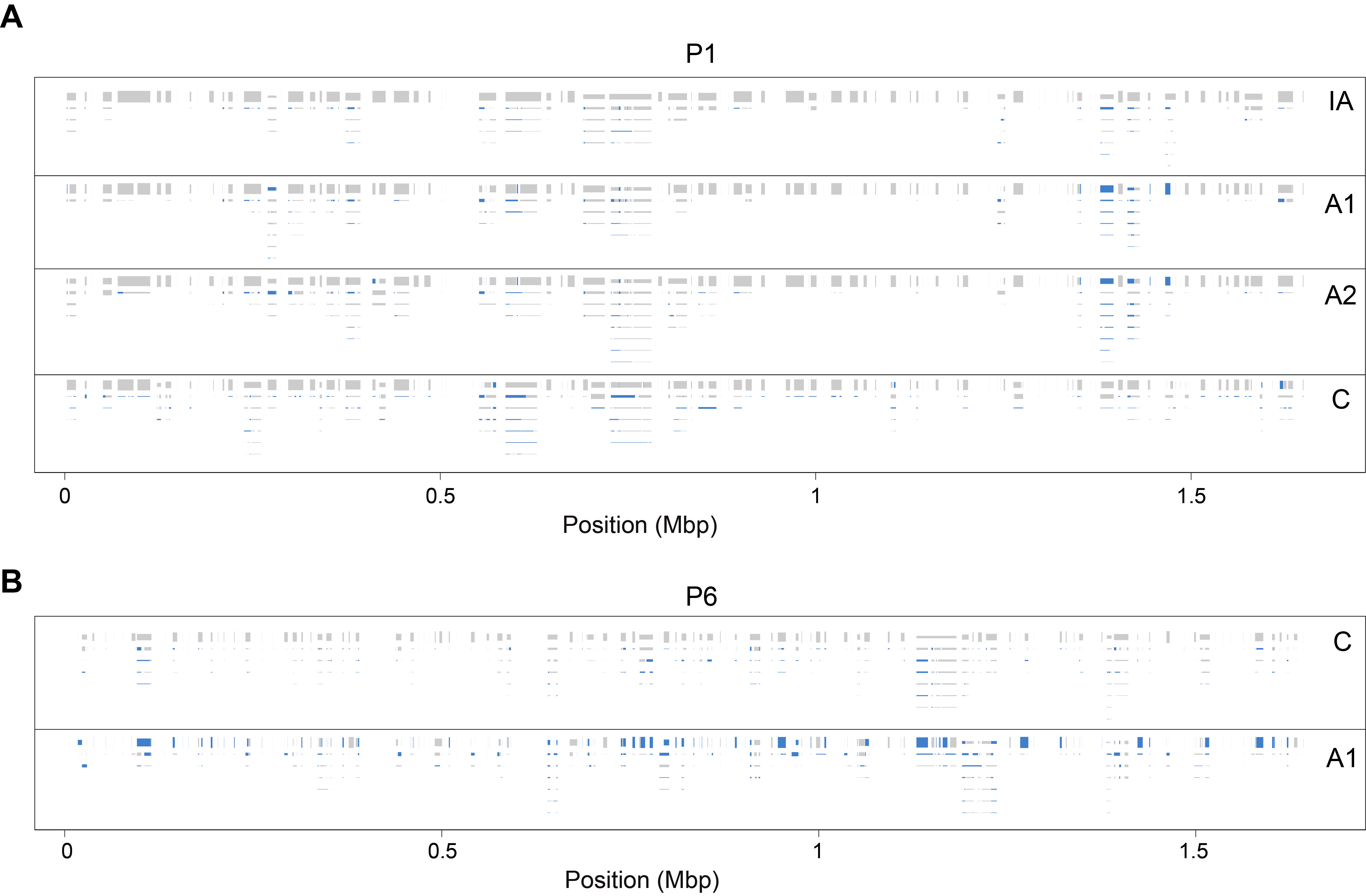


**Fig. S6. Genome-wide recombination-chunk profiles for patients P1 and P6.** (A) Genome-wide recombination-chunk profiles for IA, A1, A2, and C isolates from patient P1. Grey segments indicate regions consistent with the reference haplotype, whereas blue segments indicate recombination chunks carrying sequence variation relative to the reference. (B) Genome-wide recombination-chunk profiles for A1 and C isolates from patient P6. Grey segments indicate regions consistent with the reference haplotype, whereas blue segments indicate recombination chunks carrying sequence variation relative to the reference.


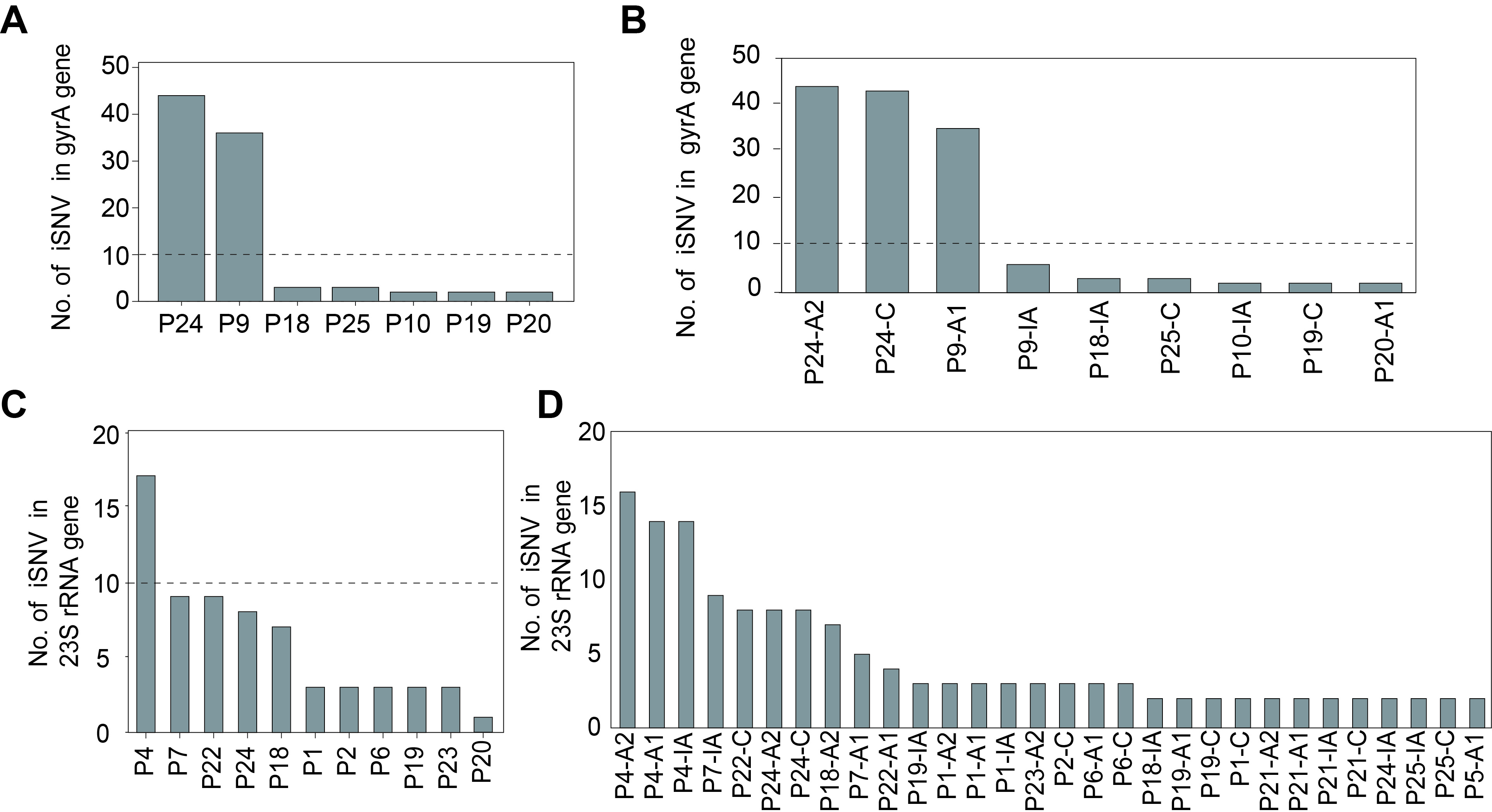


**Fig. S7. Numbers of iSNVs in levofloxacin- and clarithromycin-resistance loci.** (A–B) Numbers of iSNVs in gyrA (associated with levofloxacin resistance), summarized by patient (A) and by isolate (B). The dotted line indicates 10 iSNVs. (C–D) Numbers of iSNVs in 23S rRNA (associated with clarithromycin resistance), summarized by patient (C) and by isolate (D). The dotted line indicates 10 iSNVs.


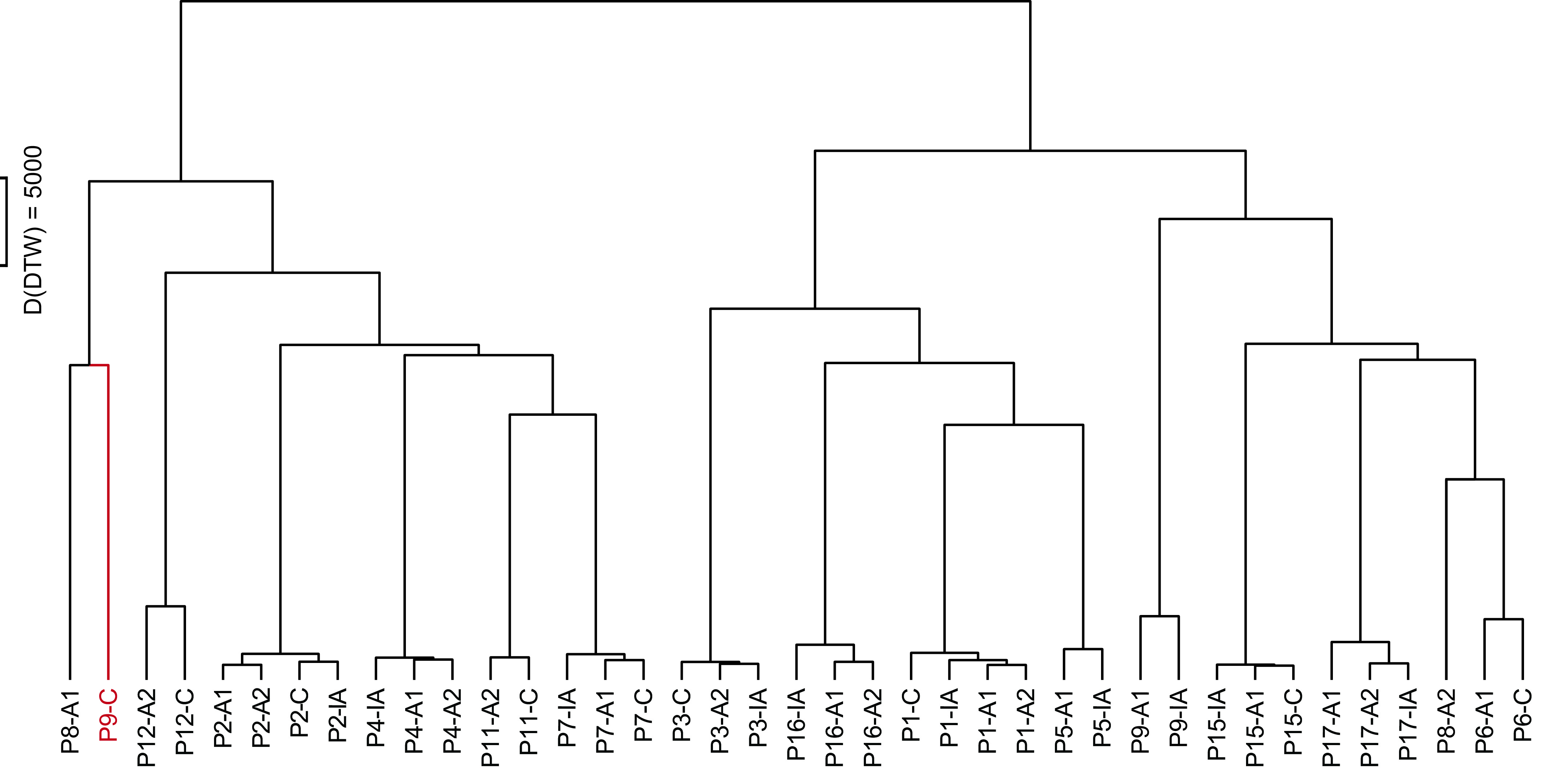


**Fig. S8. Genome-wide 5-methylcytosine (5mC) profiles of 39 *H. pylori* isolates.** Hierarchical clustering based on genome-wide 5mC profiles of 39 isolates. The red branch indicates isolate P9-C.


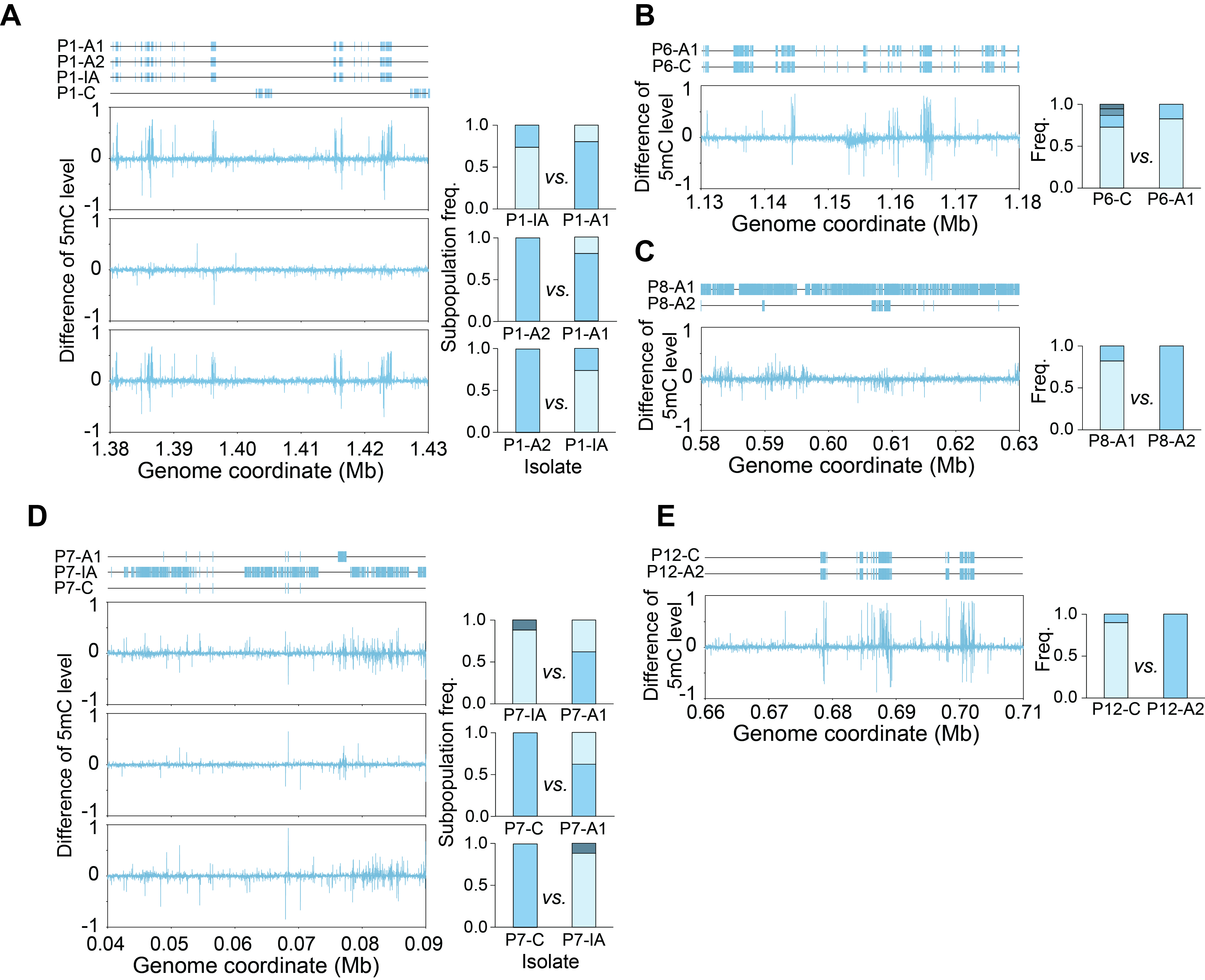


**Fig. S9. Recombinant genomic regions show differential 5mC levels.** (A) Example from patient P1. Top left, the 5-kb genomic window with the highest SNV density. Bottom left, the corresponding profile of differential 5mC levels between isolates. Right, inferred subpopulation composition across gastric regions. (B–E) Additional examples from patients P6, P7, and P12. For each patient, the top left panel shows the 5-kb window with the highest SNV density, the bottom left panel shows differential 5mC levels between isolates, and the right panel shows inferred subpopulation composition across gastric regions. Across these examples, larger differences in subpopulation composition between gastric regions are accompanied by larger-amplitude differences in 5mC levels.
