# Supplementary figures and images for: Within-host diversity and phased variant analysis reveal structures and recombination of Helicobacter pylori subpopulations in stomach
Source: Gigascience. 2026 Apr 16;15:giag046. doi: 10.1093/gigascience/giag046 (PMC13188224; doi:10.1093/gigascience/giag046)

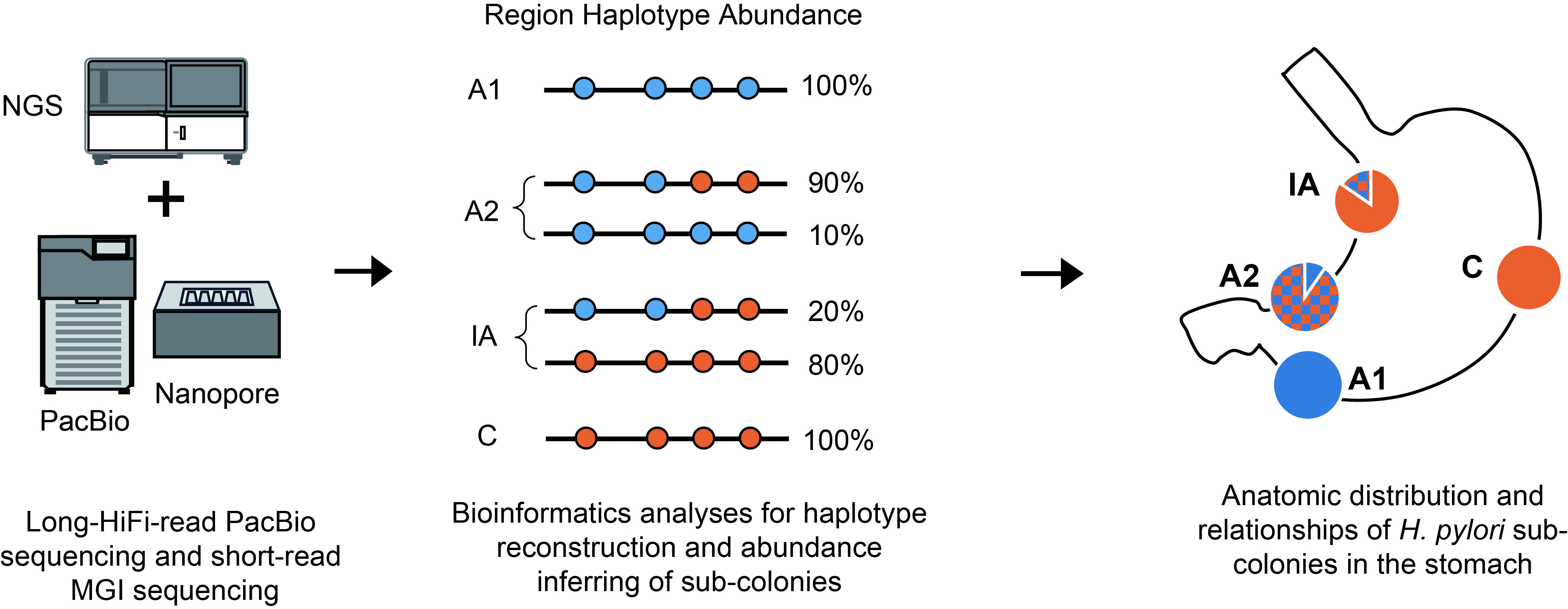

Supplement: giag046_Supplemental_Files [file giag046_supplemental_files.zip › supplementary figure/Figure S1.jpg]

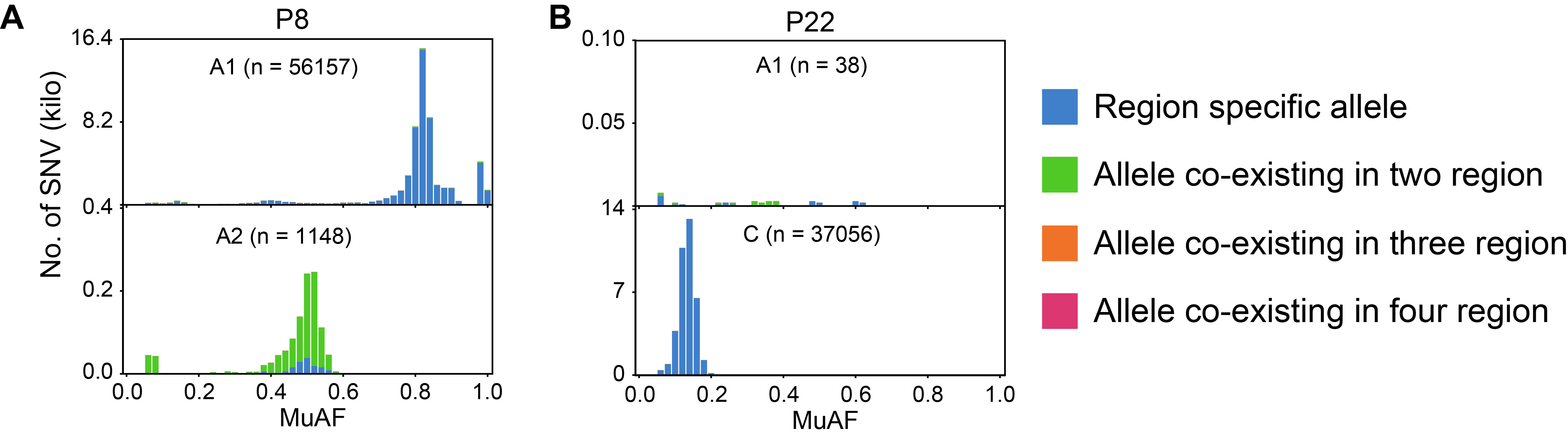

Supplement: giag046_Supplemental_Files [file giag046_supplemental_files.zip › supplementary figure/Figure S2.jpg]

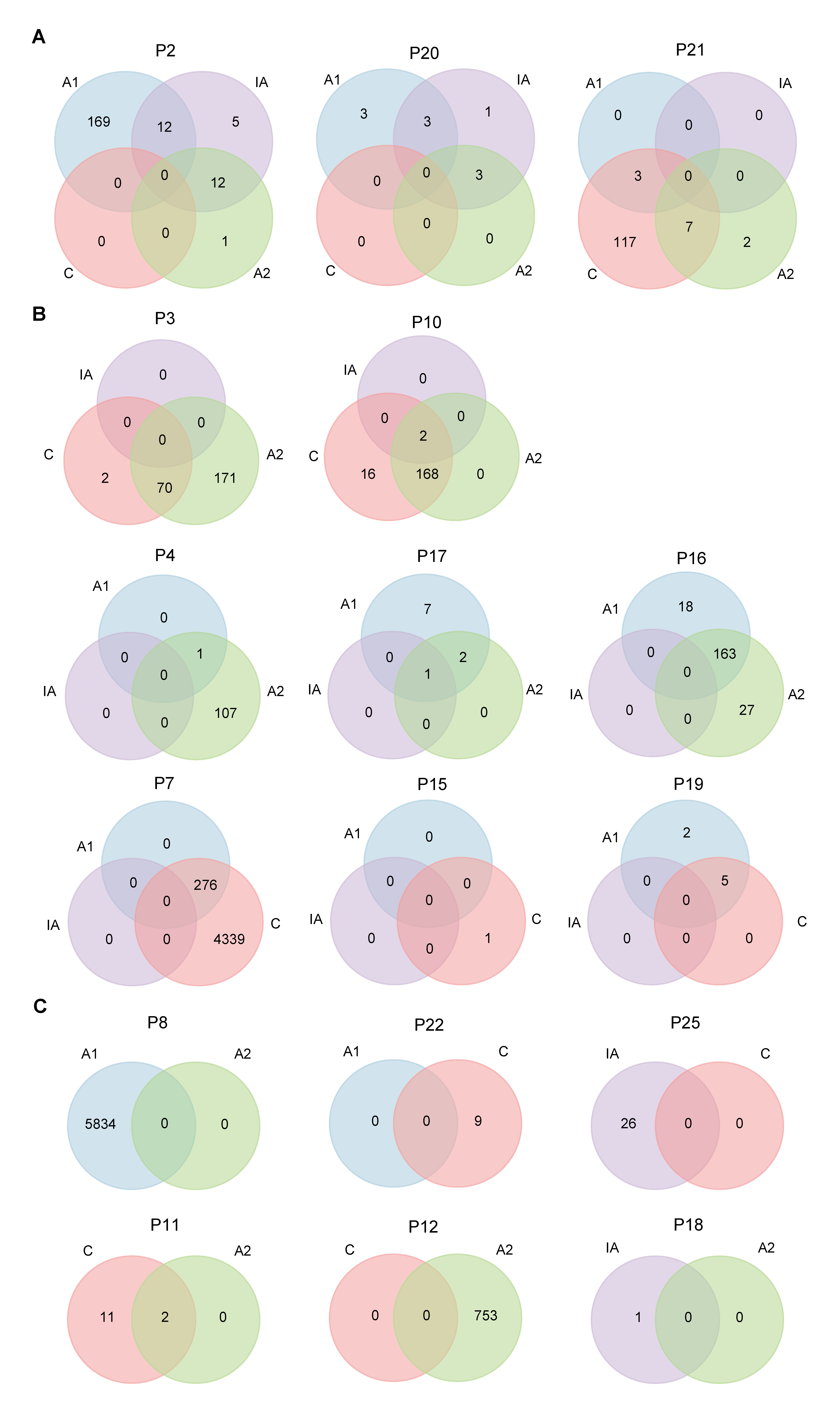

Supplement: giag046_Supplemental_Files [file giag046_supplemental_files.zip › supplementary figure/Figure S3.jpg]

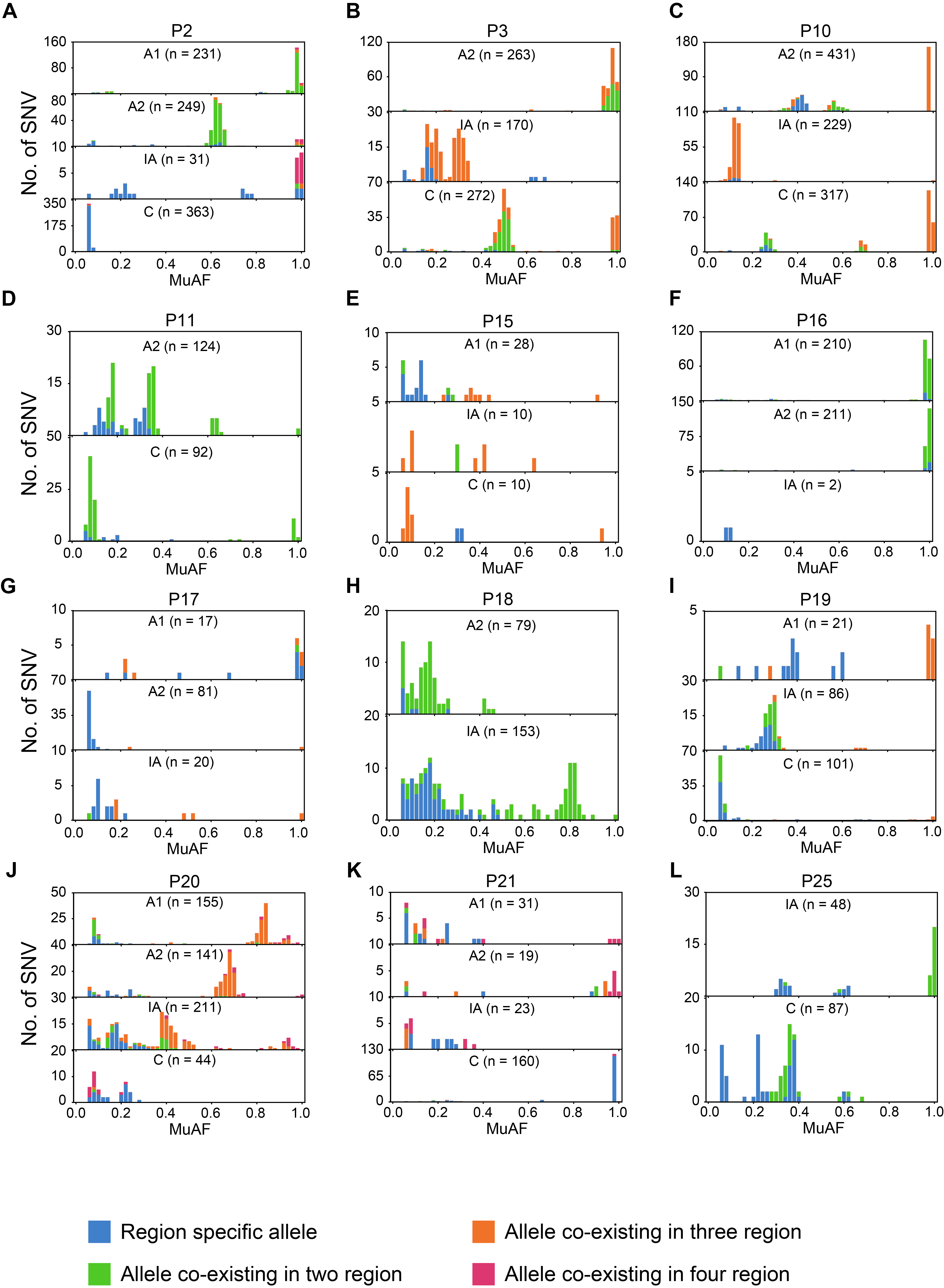

Supplement: giag046_Supplemental_Files [file giag046_supplemental_files.zip › supplementary figure/Figure S4.jpg]

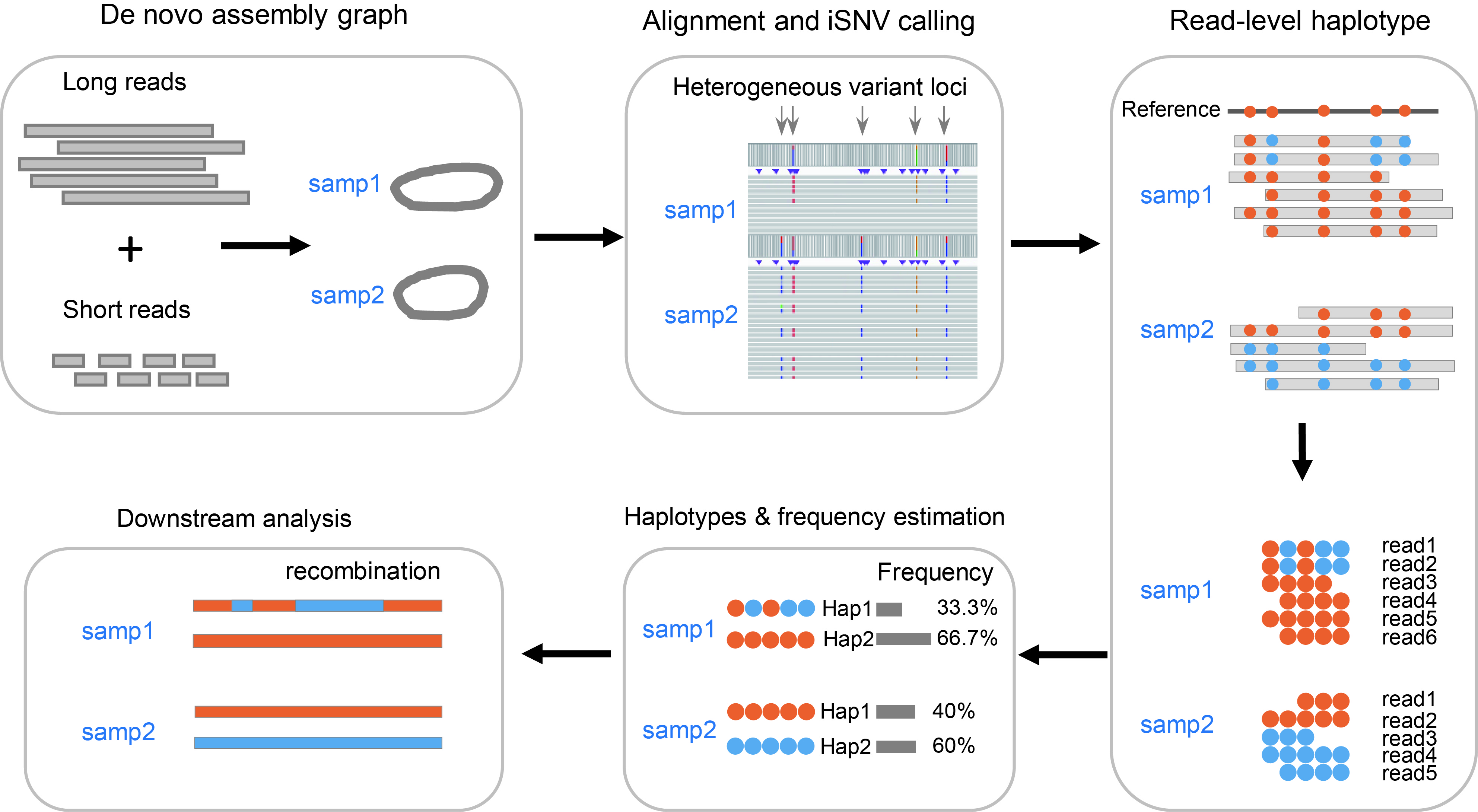

Supplement: giag046_Supplemental_Files [file giag046_supplemental_files.zip › supplementary figure/Figure S5.jpg]

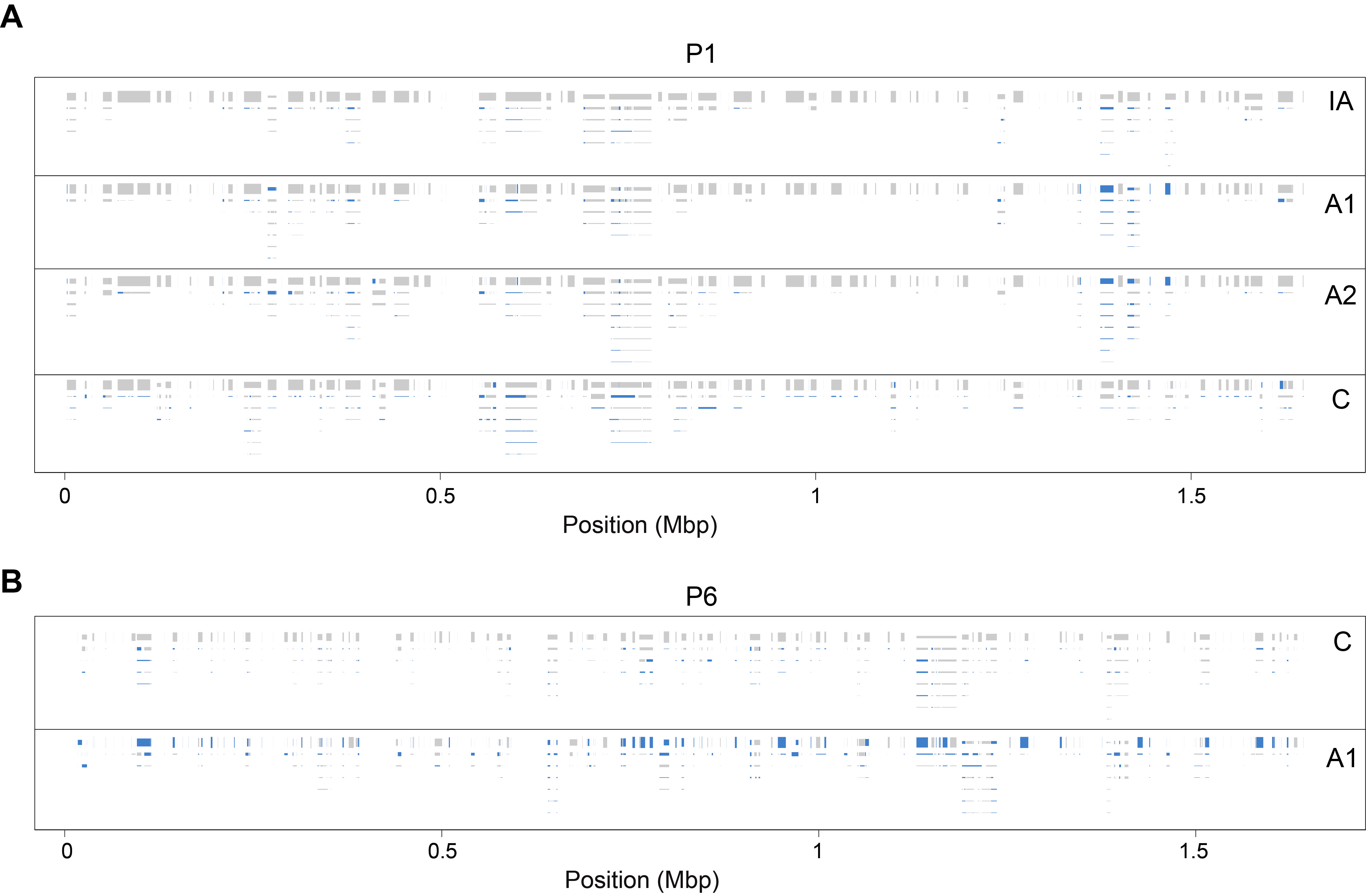

Supplement: giag046_Supplemental_Files [file giag046_supplemental_files.zip › supplementary figure/Figure S6.jpg]

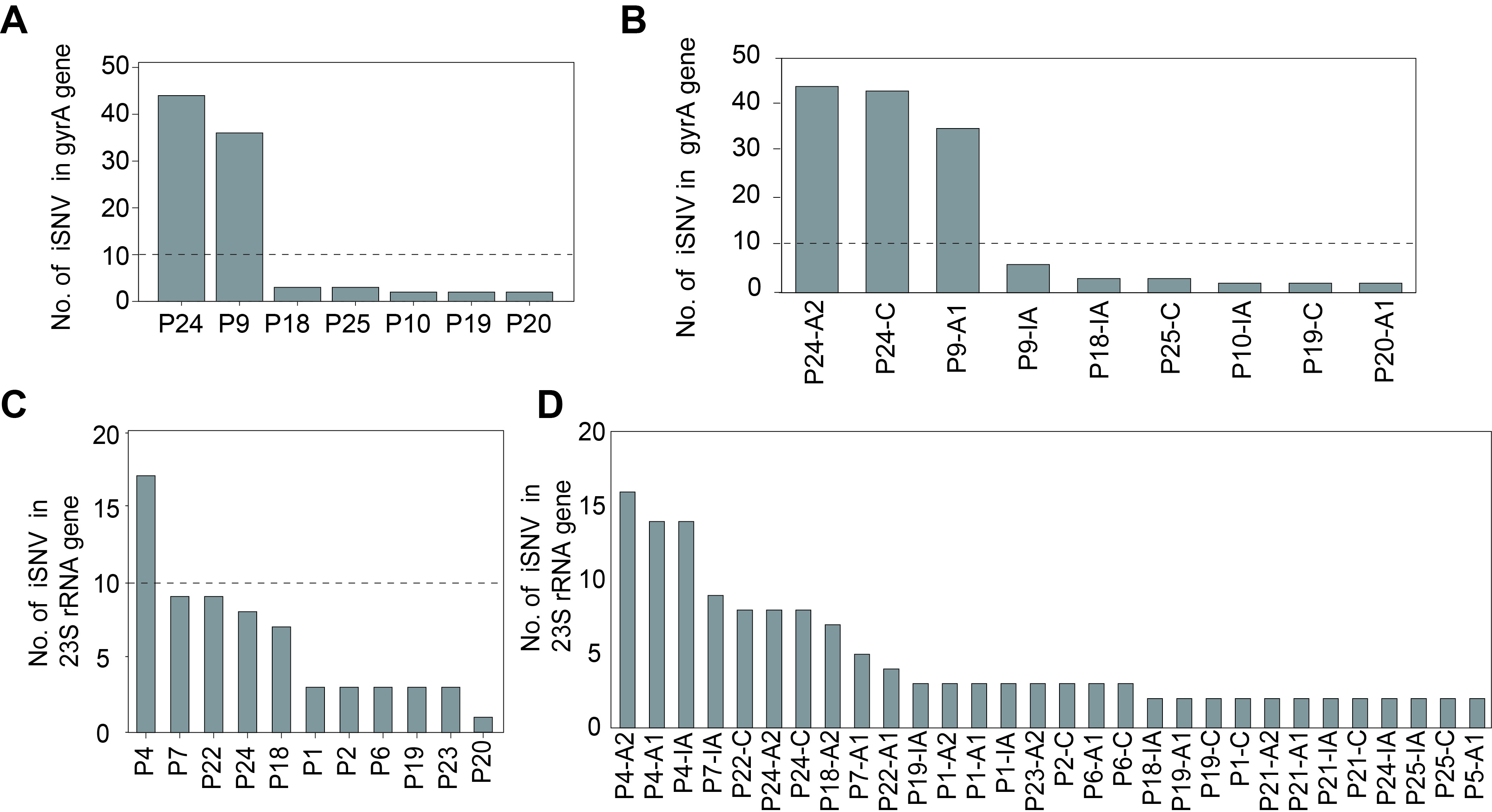

Supplement: giag046_Supplemental_Files [file giag046_supplemental_files.zip › supplementary figure/Figure S7.jpg]

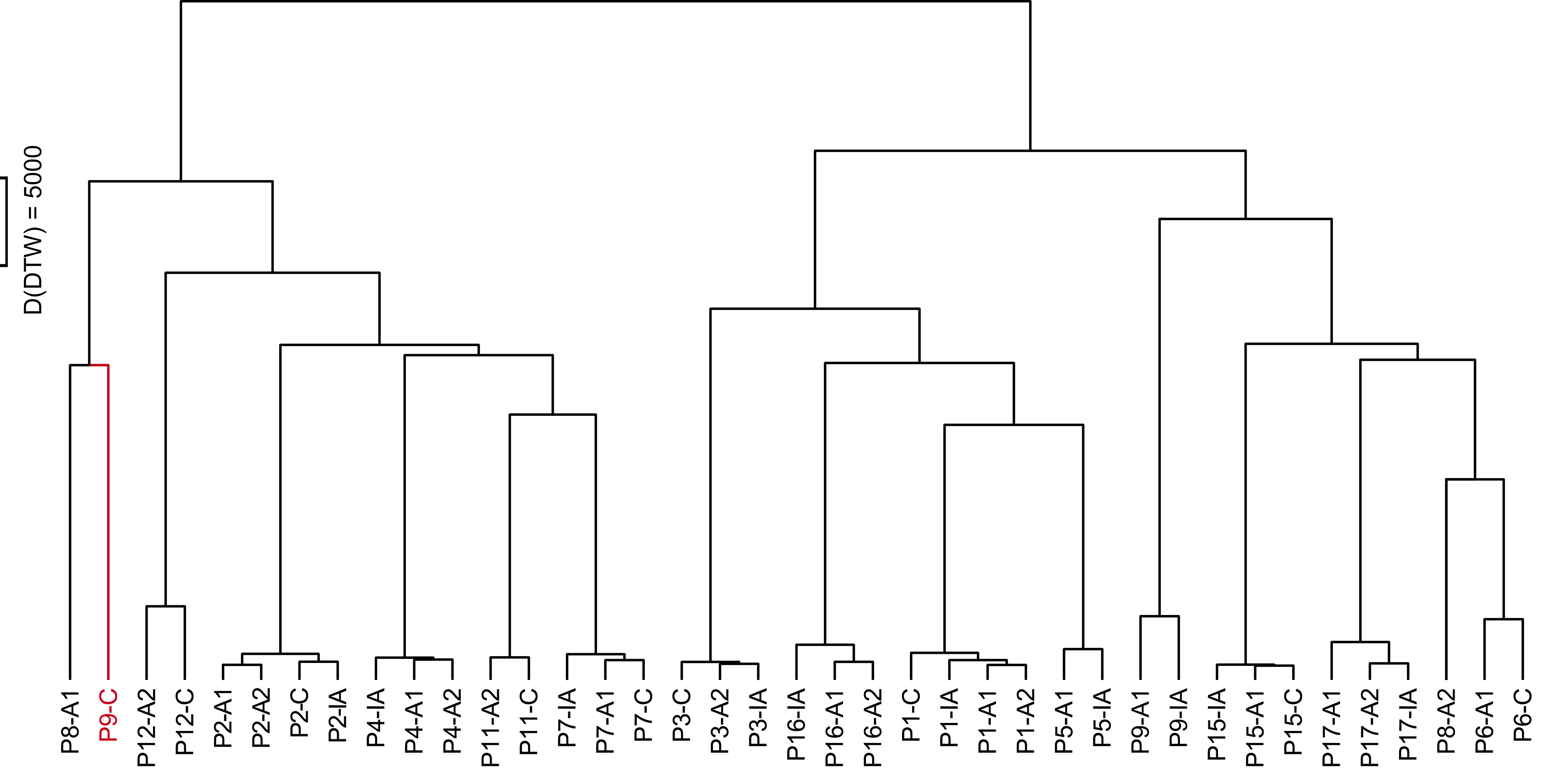

Supplement: giag046_Supplemental_Files [file giag046_supplemental_files.zip › supplementary figure/Figure S8.jpg]

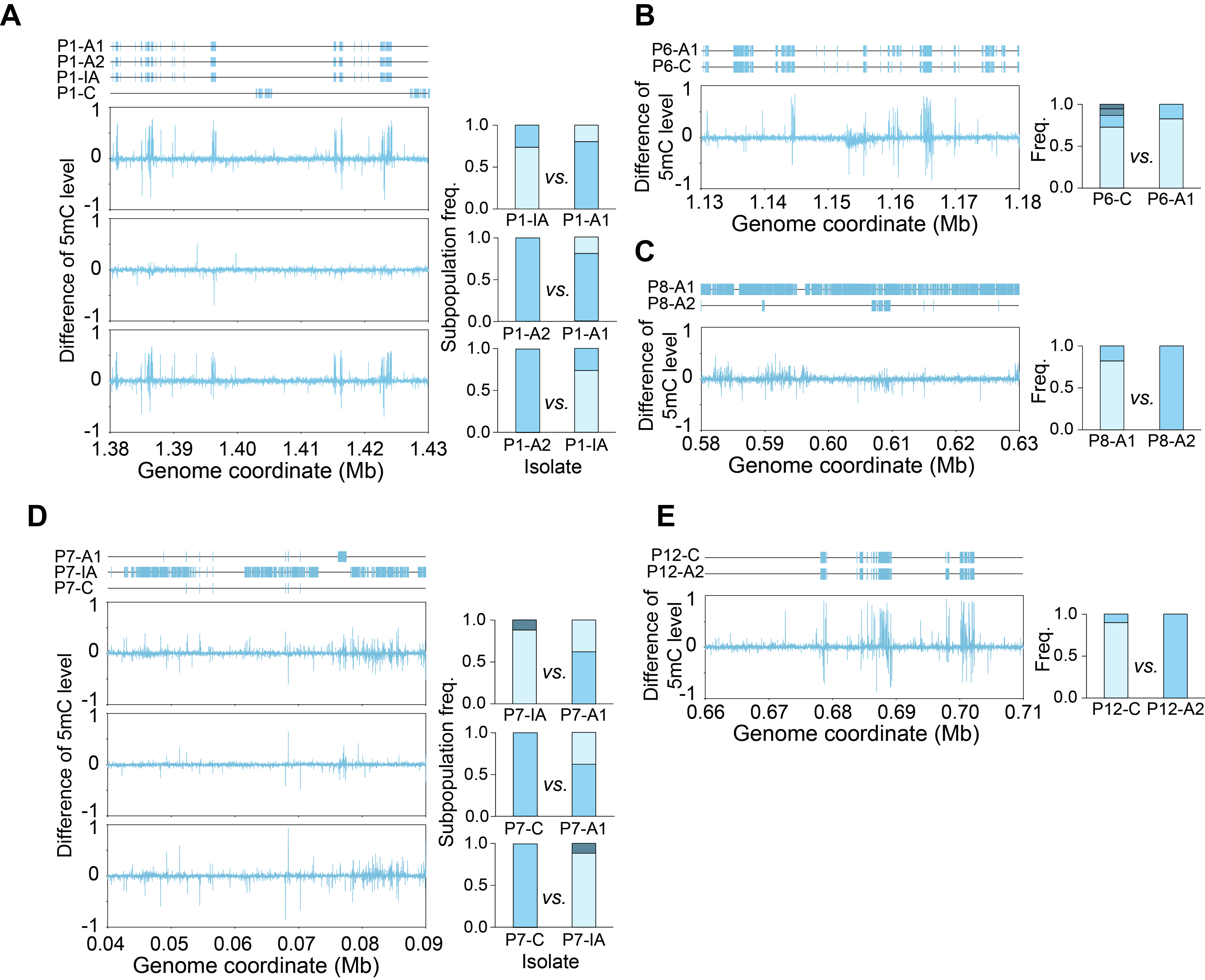

Supplement: giag046_Supplemental_Files [file giag046_supplemental_files.zip › supplementary figure/Figure S9.jpg]
