# Supplementary material for: Within-host diversity and phased variant analysis reveal structures and recombination of Helicobacter pylori subpopulations in stomach
Source: Gigascience. 2026 Apr 16;15:giag046. doi: 10.1093/gigascience/giag046 (PMC13188224; doi:10.1093/gigascience/giag046)
Supplement: giag046_GIGA-D-26-00029_original_submission [file giag046_giga-d-26-00029_original_submission.pdf]

GigaScience

Within-host diversity and phased variant analysis reveal structures and recombination of Helicobacter pylori subpopulations in stomach

--Manuscript Draft--

**Manuscript Number:**	GIGA-D-26-00029
**Full Title:**	Within-host diversity and phased variant analysis reveal structures and recombination of Helicobacter pylori subpopulations in stomach
**Article Type:**	Research
**Funding Information:**	National Natural Science Foundation of China (31870079) — Not applicable
**Abstract:**	Helicobacter pylori (H. pylori) has a highly plastic genome and can generate substantial within-host diversity during chronic gastric colonization. However, the delineation of its within-host subpopulations, particularly regarding the emergence and spread of antibiotic resistance-conferring mutations, remains poorly understood. In this study, we enrolled 25 chronic gastritis patients from southern China, collecting multiple isolates from distinct gastric regions. Among them, 14 patients exhibited heterogeneity in antibiotic susceptibility across isolates (heteroresistant), while the remaining 11 showed consistent profiles (homoresistant). Using ultra-deep short- and long-read sequencing, we showed that co-existing H. pylori subpopulations were prevalent in these patients, particularly within the same anatomical niche. Two patients presented mixed infections involving different strains as subpopulations, while others exhibited microevolution from a common ancestor. We reconstructed the subpopulation structures and found that isolates from heteroresistant patients had greater within-host diversity compared to these from homoresistant patients. Notably, subpopulations in the antrum demonstrated higher diversity than those in the gastric corpus and incisura angularis. Through a custom-developed phasing bioinformatics workflow, we resolved subpopulation-level genomic regions and directly observed extensive homologous recombination among them. Importantly, we traced the distribution of levofloxacin- and clarithromycin-associated resistance mutations across subpopulations, which was mainly mediated by recombination. To our knowledge, this study provides the first detailed depiction of H. pylori subpopulation distribution within the human stomach, illustrating how recombination drives within-host diversification and contributed to the spread of antibiotic resistance mutations.
**Corresponding Author:**	Ming Ni<br>Academy of Military Medical Sciences<br>Beijing, CHINA
**Corresponding Author Secondary Information:**	
**Corresponding Author's Institution:**	Academy of Military Medical Sciences
**Corresponding Author's Secondary Institution:**	
**First Author:**	Xiaomin Zhang
**First Author Secondary Information:**	
**Order of Authors:**	Xiaomin Zhang<br>Hongjie Liu<br>Siyue Xu<br>Shuang Zhang<br>Tingting Yang<br>Zhongyi Lei<br>Weili Xu

Powered by Editorial Manager® and ProduXion Manager® from Aries Systems Corporation

	Xiaochen Bo
	Chenghai Yang
	Ming Ni
Order of Authors Secondary Information:	
Additional Information:	
Question	Response
Are you submitting this manuscript to a special series or article collection?	No
**Experimental design and statistics**<br><br>Full details of the experimental design and statistical methods used should be given in the Methods section, as detailed in our Minimum Standards Reporting Checklist. Information essential to interpreting the data presented should be made available in the figure legends.<br><br>Have you included all the information requested in your manuscript?	Yes
**Resources**<br><br>A description of all resources used, including antibodies, cell lines, animals and software tools, with enough information to allow them to be uniquely identified, should be included in the Methods section. Authors are strongly encouraged to cite Research Resource Identifiers (RRIDs) for antibodies, model organisms and tools, where possible.<br><br>Have you included the information requested as detailed in our Minimum Standards Reporting Checklist?	Yes
**Availability of data and materials**<br><br>All datasets and code on which the conclusions of the paper rely must be either included in your submission or deposited in publicly available repositories (where available and ethically	Yes

appropriate), referencing such data using a unique identifier in the references and in the “Availability of Data and Materials” section of your manuscript.<br><br>Have you have met the above requirement as detailed in our [Minimum Standards Reporting Checklist](#)?	
GigaScience has policies and guidelines in place for the use of generative AI-writing tools such as ChatGPT. If you have used such writing tools to assist with writing the manuscript this must be declared and cited in the text. Authors should not list AI-writing tools and other AI-assisted technologies as an author or co-author and should acknowledge that they are fully responsible for text generated or refined by AI-writing tools.<p><br><br>A summary of use (particularly in the introduction or among methods) needs to be included at the end of the paper, and the outputs should also be included as a supplementary file hosted in GigaDB or other open repositories. Please read our guidelines for more information. <p><br><br>By submitting to GigaScience, you are aware of the journal's AI-writing tools policy, and if you have declared use of such tools below, you have acknowledged this where appropriate in your manuscript and have made a summary of use and outputs available. <p><br><b>AI-assisted writing tools have been used in the preparation of this manuscript?	No

Manuscript Click here to access/download;Manuscript;Main Manuscript.docx

Within-host diversity and phased variant analysis reveal structures and recombination of *Helicobacter pylori* subpopulations in stomach

Authors: Xiaomin Zhang[1†], Hongjie Liu[2,3†], Siyue Xu[5†], Shuang Zhang[2], Tingting Yang[1,6], Zhongyi Lei[1,7], Weili Xu[8], Xiaochen Bo[1], Chenghai Yang[4], Ming Ni[1*]

Affiliations:

[1] Academy of Military Medical Sciences, Beijing, China.

[2] Shanghai Key Laboratory of Tuberculosis, Shanghai Pulmonary Hospital, Shanghai, China.

[3] Tongji University School of Medicine, Shanghai, China.

[4] Integrative Clinical Microecology Center, Shenzhen Key Laboratory of Gastrointestinal Microbiota and Disease, Department of Gastroenterology, Shenzhen Hospital, Southern Medical University, Shenzhen, China.

[5] Youran Digital Intelligence (SUZHOU) Medical Technology. CO., LTD., Suzhou, China.

[6] School of Forensic Medicine, Shanxi Medical University, Taiyuan, People's Republic of China.

[7] College of Life Science and Technology, Beijing University of Chemical Technology, Beijing, China.

[8] Hangzhou Zhiyuan Medical Laboratory Co., Ltd., Hangzhou, China.

[†]Co-author: These authors contributed equally to this work.

[*]Correspondence: M Ni, niming@bmi.ac.cn.

Abstract

Helicobacter pylori (*H. pylori*) has a highly plastic genome and can generate substantial within-host diversity during chronic gastric colonization. However, the delineation of its within-host subpopulations, particularly regarding the emergence and spread of antibiotic resistance-conferring mutations, remains poorly understood. In this study, we enrolled 25 chronic gastritis patients from southern China, collecting multiple isolates from distinct gastric regions. Among them, 14 patients exhibited heterogeneity in antibiotic susceptibility across isolates (heteroresistant), while the remaining 11 showed consistent profiles (homoresistant). Using ultra-deep short- and long-read sequencing, we showed that co-existing *H. pylori* subpopulations were prevalent in these patients, particularly within the same anatomical niche. Two patients presented mixed infections involving different strains as subpopulations, while others exhibited microevolution from a common ancestor. We reconstructed the subpopulation structures and found that isolates from heteroresistant patients had greater within-host diversity compared to these from homoresistant patients. Notably, subpopulations in the antrum demonstrated higher diversity than those in the gastric corpus and incisura angularis. Through a custom-developed phasing bioinformatics workflow, we resolved subpopulation-level genomic regions and directly observed extensive homologous recombination among them. Importantly, we traced the distribution of levofloxacin- and clarithromycin-associated resistance mutations across subpopulations, which was mainly mediated by recombination. To our knowledge, this study provides the first detailed depiction of *H. pylori* subpopulation distribution within the human stomach, illustrating how recombination drives within-host diversification and contributed to the spread of antibiotic resistance mutations.

Introduction

Helicobacter pylori (*H. pylori*) is a pathogen that colonizes the human stomach[1, 2]and is estimated to infect more than 40% of the global population[3, 4]. *H. pylori* infection underlies gastritis, gastric and duodenal ulcers, and gastric cancer[1, 5-8]. In 1994, the International Agency for Research on Cancer classified *H. pylori* infection as carcinogenic to humans (Group 1) for non-cardia gastric cancer[9]. Eradication therapy reduces the incidence of gastric cancer[10, 11], and proton pump inhibitors (PPIs) and antibiotics are widely used[1, 12]. However, eradication rates have declined worldwide, because antibiotic resistance is a major cause of treatment failure[12, 13].

The rise of antibiotic-resistant *H. pylori* reflects its highly plastic genome and rapid adaptive capacity during chronic infection. As a naturally competent bacterium[14], *H. pylori* undergo DNA transformation at rates far exceeding those of *Escherichia coli* (*E. coli*) and *Bacillus subtilis*[15, 16]. Homologous recombination is a major driver of genetic diversity[16, 17]; it acts across the ~1.6 Mb genome and can introduce variation several- to hundreds-fold greater than mutation[16, 17]. In addition, *H. pylori* lack a canonical mismatch-repair system[18, 19], yielding mutation rates of 10^{-5} to 10^{-6} per site per year[20, 21], which are 10–100 times higher than those measured for *E. coli*[22, 23].

Consequently, *H. pylori* can exhibit substantial within-host heterogeneity during chronic infection. Antibiotic heteroresistance refers to the coexistence of resistant and susceptible subpopulations within the same infection, representing a transitional stage from susceptibility to resistance and leading to reduced therapeutic efficacy[24]. However, a single strain isolated from one biopsy may not be representative, especially for antibiotic susceptibility testing[25, 26]. Longitudinal studies also show continual remodeling of within-host populations by mutation and homologous recombination

over the course of chronic infection[16, 17, 27, 28]. Notably, Ailloud and colleagues sampled several gastric regions and sequenced ~10 single colonies per biopsy by whole-genome sequencing, demonstrating multiclonality within a single stomach[29]. Despite these advances, how resistance-conferring mutations emerge and disseminate among coexisting subpopulations across gastric niches remains incompletely resolved.

Sampling from several gastric regions with colony isolation and whole-genome sequencing can reveal multiclonality and migration across anatomical regions, but this approach is labor-intensive and costly[29]. Population deep sequencing provides a complementary alternative that detects mixed-lineage signals from a single specimen using allele-frequency profiles and clusters of nucleotide polymorphisms. However, it remains challenging to resolve subpopulations and trace recombinant segments. Tools for genomic variant phased (haplotype) analysis, including WhatsHap[30], HapCUT2[31], FALCON[32], hifiasm[33], and DipAsm[34] are for diploid genomes, and cannot be applied to reconstruct within-host haplotypes of *H. pylori* within-host subpopulations.

In this study, we collected *H. pylori* isolates from multiple gastric regions of chronic gastritis patients from southern China. Using ultra-deep long-read and short-read sequencing and a custom bioinformatics phasing workflow, we reconstructed the subpopulation structure and, thereby, revealed the relationships, relative abundances, as well as the recombination dynamics among these subpopulations.

Results

Clinical characteristics of patients and genomic features of *H. pylori* isolates

Twenty-five patients with chronic gastritis were enrolled. For each patient, two to four *H. pylori* isolates were obtained from different gastric regions, including the

greater curvature of the antrum (A1), lesser curvature of the antrum (A2), incisura angularis (IA), and corpus of the stomach (C). Based on the antibiotic susceptibility of their *H. pylori* isolates, the patients were divided into a heteroresistance group (n = 14, P1-P14) and a homoresistance group (n = 11, P15-P25). As shown in **Fig. 1A**, isolates from patients in the heteroresistance group exhibited diverse susceptibility to levofloxacin and/or clarithromycin among gastric regions, whereas those from the homoresistance group displayed consistent susceptibility. In total, 69 isolates were obtained and indexed by patient and gastric region. For example, P1-A1 denotes the isolate was from the greater curvature of the antrum (A1) of patient P1. The clinical characteristics of the patients and the antibiotic susceptibility of the *H. pylori* isolates are summarized in **Table 1**.

Table 1 The characteristics of patients and antibiotic susceptibility testing of *H. pylori* isolates. *H. pylori* isolates were from multiple gastric regions including antrum (A1), lesser curvature of the antrum (A2), incisura angularis (IA), and corpus of the stomach (C). R, resistant isolate; S, susceptible isolate.

Group	Patient	Gender	Age (y)	Clinical diagnosis	Susceptibility to clarithromycin				Susceptibility to levofloxacin			
					A2	A1	IA	C	A2	A1	IA	C
Heteroresistance group (*n*=14)	P1	Female	53	chronic gastritis	S	R	S	R	S	S	S	S
	P2	Male	31	chronic gastritis	S	S	S	R	S	S	S	R
	P3	Female	30	chronic gastritis	S	-	R	S	S	-	R	S
	P4	Male	52	chronic gastritis	R	S	R	-	S	S	S	-
	P5	Male	43	chronic gastritis	-	S	R	-	-	S	R	-
	P6	Male	52	chronic gastritis	-	R	-	S	-	R	-	S
	P7	Male	36	chronic gastritis	-	R	R	R	-	S	R	R
	P8	Female	48	chronic gastritis	S	S	-	S	S	S	-	R
	P9	Male	42	chronic gastritis	-	S	R	R	-	S	R	R
	P10	Male	41	-	R	-	R	R	R	-	S	R
	P11	Male	45	chronic gastritis	S	-	R	R	S	-	R	R
	P12	Male	36	chronic gastritis	R	-	-	S	S	-	-	S
	P13	Female	42	chronic gastritis, duodenal bulb ulcer	R	S	S	-	R	R	R	-
	P14	Male	34	chronic gastritis	S	R	S	-	S	R	R	-
Homoresistance group (*n*=11)	P15	Male	51	chronic gastritis	-	S	S	S	-	S	S	S
	P16	Male	53	chronic gastritis	S	S	S	-	S	S	S	-
	P17	Male	58	chronic gastritis	R	R	R	-	R	R	R	-
	P18	Male	48	chronic gastritis	R	-	R	-	R	-	R	-
	P19	Male	50	chronic gastritis with antral ulcer	-	R	R	R	-	R	R	R
	P20	Male	34	chronic gastritis	R	R	R	R	R	R	R	R
	P21	Female	37	chronic gastritis	S	S	S	S	R	R	R	R
	P22	Male	62	chronic gastritis	-	S	-	S	-	R	-	R
	P23	Male	48	chronic gastritis	S	-	S	-	R	-	R	-
	P24	Male	39	chronic gastritis with incisura angularis ulcer	R	-	R	R	S	-	S	S
	P25	Female	38	chronic gastritis	-	-	R	R	-	-	S	S

All *H. pylori* isolates were subjected to ultra-deep short-read and long-read

sequencing (**Fig. S1**). The genome assemblies had an average length of 1.61±0.03 Mbp and a GC content of 38.7± 0.08% (**Table S1**). All these genome assemblies carried a single copy of the virulence gene *cagA*. A comparison with 233 *H. pylori* reference strains from known global lineages (**Table S2**) showed that all genomes, except P8-A1, belonged to the hspEAsia lineage, including P8-A2 (**Fig. 1B**). This pattern indicates that P8 had mixed infection with two distinct *H. pylori* strains.

The maximum likelihood phylogeny was reconstructed from the core genomes of the *H. pylori* 69 isolates (0.76 Mbp, 816 genes) together with the reference strain Puno135 (GenBank accession NC_017379.1). The tree was further adjusted for homologous recombination using ClonalFrame[35]. Except P8, isolates from the same host clustered tightly. These results are consistent with previous reports on within-host evolution and mixed infection of *H. pylori* [29, 36], suggesting that the observed intra-host differentiation of *Helicobacter pylori* primarily arises from microevolution within the human gastric niche. Notably, although P8-A1 and P8-A2 were assigned to different global lineages, they fell on the same branch of the reconstructed phylogeny, indicating genetic exchange between the *H. pylori* sub-colonies within the host.

Antibiotic heteroresistant patients show higher within-host *H. pylori* diversity

Since the core genome had a limited size (0.76 Mbp), patient-specific near-complete genomes (~1.61 Mbp) were used as the corresponding reference for each patient to characterize genome-wide diversity. P13 and P14 were excluded due to the absence of complete genome assemblies, resulting from the failure of long-read sequencing. For the rest patients, full-genome-wide single nucleotide variations (SNVs) of their *H. pylori* isolates were identified, excluding loci in repetitive regions.

We found prevalent heterozygous SNVs in these *H. pylori* isolates. Most of SNVs

were bi-allelic, and 85.4% (121,395 of 142,206) had mutated alleles with a supporting read frequency (MuAFs) ranged from 0.05 to 0.95. In the following analyses, we classified SNVs with one dominant allele (MuAF ≥ 0.95) as single nucleotide polymorphisms (SNPs), while those with a MuAF between 0.05 and 0.95 were termed intra-region single nucleotide variations (iSNVs), as illustrated in **Fig. 2A**. Namely, for a given patient, SNPs reflect the intra-host divergence of the *H. pylori* isolates among gastric regions, and iSNVs could capture subtle within-stomach-region heterogeneity of *H. pylori*.

Analysis of SNPs revealed that *H. pylori* isolate from the heteroresistance group carried significantly more intra-host SNP loci than those from the homoresistance group (two-sided Wilcoxon rank-sum test, P = 0.0003; **Fig. 2B**). Among heteroresistant patients, the number of intra-host SNP loci ranged from 13 to 5,834 (mean = 1,701; median = 404), with four patients harboring more than 3,600 SNPs. By contrast, eight of the eleven patients in homoresistant group had ten or fewer SNPs, and the maximum number was 208.

The two isolates from P8, a patient with a mixed infection, exhibited the highest number of SNP loci (n = 5,834). This count was only marginally higher than the 4,615 loci found in P7, who was infected with genetically closely related *H. pylori* strains. We further analyzed the iSNVs and found that the two strains co-existed within gastric region A1 of P8, instead of existing in A1 and A2 respectively. Isolate P8-A1 harbored 50,471 iSNVs, far exceeding the number in P8-A2 (**Fig. 2C**). Based on the distribution of MuAFs (**Fig. S2A**), we could quantify relative abundance of the two strains in P8-A1 (82% and 18%) and P8-A2 was dominated by one strain (**Fig. 2D**). The dominant strain in A1 differed from that in A2.

Interestingly, using the iSNV-based analysis, we identified another patient, P22,

also with mixed infection of distinct *H. pylori* strains. P22-C contained 37,152 iSNVs, indicating the presence of other strain as a sub-colony accounting for 14% of the population in C region (**Fig. 2C** and **Fig. S2B**). P22-A1 was dominated by the dominant strain of P22-C. Consequently, the mixed-infection of P22 were failed to be detected via SNP-based analysis (**Fig. 2E**).

The remaining patients were more likely to be infected by a single ancestral *H. pylori* strain that later diversified within the host, and the isolates in the heteroresistance group still tended to harbor more iSNVs than those in the homoresistance group. However, the difference was not statistically significant (two-sided Wilcoxon rank-sum test, P = 0.084; **Fig. 2F**). It was largely due to the high iSNV counts in two homoresistant patients P23 and P24. Notably, in many cases, the number of iSNVs and SNPs were inconsistent. Beside P24 and P23, P1, P2, P4 and P6 from heteroresistance group also exhibited remarkably more iSNVs (n > 3000) than SNPs (n < 400). Patient P5 had 3,685 SNPs but only 225 iSNVs. Taken together, these findings suggest that, relative to using SNPs alone, iSNVs could provide a more comprehensive and nuanced view of how *H. pylori* subpopulations distributed within the stomach.

Coexistence of within-host *H. pylori* subpopulations across gastric regions

Next, to explore the migration and diversification of *H. pylori* within the host, we analyzed the distribution and relationships of subpopulations across different gastric regions in the 21 patients without mixed infections (i.e., excluding P8 and P22). We conducted the analyses based on MuAF distributions and sharing of SNVs among isolates from the identical patient. In eight patients, their *H. pylori* isolates harbored ≥500 within-host iSNV loci, which were sufficient for subpopulation inferring.

Six (P4, P5, P7, P12, P23, and P24) of the eight patients exhibited clear evidence of two main coexisting subpopulations. In these cases, the majority of SNVs were shared across

regions, and their MuAFs centered in one peak in their distributions, indicating the presence of two subpopulations with varying relative abundances across gastric regions (**Fig. 3A–F**). The inferred subpopulation structures of the 14 isolates from the six patients are illustrated in **Fig. 3G**. Among them, ten isolates contained two coexisting subpopulations, and the other four were dominated by a single population (P5-IA, P7-C, P12-A2, P23-A1). The minor and dominant subpopulations could switch roles across gastric regions. For example, the minor subpopulations in P5-A1 and P12-C became dominant in P5-IA and P12-A2, respectively. All the six patients carried no more than two within-host subpopulations of *H. pylori*, with the exception of P7. In P7-IA, we identified an additional subpopulation marked by region-specific iSNVs, suggesting the emergence of a third subpopulation.

Notably, for the 14 isolates from the six patients, *H. pylori* colonized in A1 and A2 gastric regions exhibited greater subpopulation heterogeneity than those in IA and C regions. Namely, the minor subpopulations in A1 and A2 regions tended to have significantly higher relative abundances than their counterparts in IA and C (two-sided Wilcoxon rank-sum test, $P = 0.011$; **Fig. 3H**).

Haplotype phasing reveals recombination among within-host *H. pylori* subpopulations

P1 and P6 had apparently differed SNV sharing and MuAF distribution from those in the above-mentioned six patients. In P1, SNVs of P1-A1, P1-A2 and P1-IA isolates were largely shared, while in A1 and A2, the MuAFs of SNVs exhibited two peaks in distributions (**Fig. 4A**). It implies extensive recombination occurred among the coexisting subpopulations in gastric regions. To characterize this recombination at the subpopulation level, the analysis based on separate iSNVs is not applicable. Therefore, we developed a custom bioinformatics workflow for SNV phasing and haplotype reconstruction. This workflow utilized PacBio HiFi long sequencing reads to phase SNVs and estimate their relative abundance in population (Methods; **Fig. S4**). Since the workflow required a sufficient number of SNVs, we extracted genomic regions with abundant SNVs (indicated by shaded areas in **Fig. 4B**) for the phasing analysis.

Via phasing of SNVs, we revealed the genomic homologous recombination among the *H. pylori* subpopulations within P1 (**Fig. 4C**). Notably, two clusters of SNVs near the 1.4 Mb genomic coordinates (indicated by triangles in **Fig. 4C**) exhibited diversified appearances in subpopulations (**Fig. 4A**). In detail, the two SNV clusters were phased with other clusters in the minor subpopulations in P1-IA, while in P1-A1 they exchanged into the major subpopulations. In P1-A2, they appeared in both the minor and major subpopulation. On the other hand, P1-C had >3,700 SNVs, most of which were isolate-specific and in the minor subpopulation. Haplotype phasing revealed that the minor subpopulation in P1-C underwent more extensive recombination than the other subpopulations (**Fig. S5A**).

In P6, MuAFs of SNVs in the P6-A1 isolate formed three distinct peaks, and these SNVs were largely shared with those in P6-C (**Fig. 4D**), suggesting more complicated genetic exchange among subpopulations than in P1. Phasing was performed on genomic regions enriched for SNVs (**Fig. 4E**) and revealed a diverse subpopulation structure in P6-C (**Fig. 4F**). The major subpopulation accounted for 72.7% of the population in P6-C, and three additional subpopulations coexisted with a ≥5% population frequency (from 5.5% to 13.9%, **Fig. 4F**). In P6-A1, SNVs were distributed across both the dominant and minor subpopulations and could be assigned to three groups, respectively corresponding to the three MuAF peaks observed in P6-A1 (**Fig. 4D**): the specific SNVs in the minor subpopulation (MuAF ~ 0.10), the specific SNVs in the major subpopulation (MuAF ~ 0.90), and the shared SNVs in both subpopulations (MuAF ~ 0.98).

We then conducted the phasing analysis workflow for 31 isolates with PacBio sequencing data (excluding P8 and P22, MuAF distributions provided in **Fig. S3**). With a threshold of at least six SNVs per haplotype, we identified a total of 2,177 haplotypes at the subpopulation level, which were referred to as SNV chunks (to be distinguished from SNV clusters). The median size of these SNV chunks was 2,583 bp (**Fig. 4G**).

Recombination mediates spread of antibiotic-resistance mutations across within-host subpopulations

We next investigated how antibiotic-resistance–conferring mutations arise and spread at the within-host subpopulation level. We focused on the G271A substitution in *gyrA* (leads D91N in DNA gyrAse subunit A) conferring resistance to levofloxacin (LEV), and the A2142G/A2143G substitutions in the *23S rRNA* gene conferring resistance to clarithromycin (CLA). Three patients were selected for the phasing analysis since their isolates had enriched iSNVs at the two genes (P24 and P9 for *gyrA*, P4 for *23S rRNA*, **Fig. S6**).

SNV haplotypes in *gyrA* and the *23S rRNA* gene at the subpopulation level were respectively reconstructed. In isolate P9-IA, a single subpopulation carrying the G271A mutation in *gyrA* dominated, consistent with the LEV susceptibility result (**Fig. 5A**). Interestingly, the *gyrA* haplotype in P9-IA appeared as a recombinant of the minor and major subpopulations in P9-A1, indicating that the mutation in P9-IA was derived from recombination. For P24, both P24-A2 and P24-C were susceptible to LEV. However, we found that both P24-A2 and P24-C carried the G271A mutation in *gyrA*, respectively in the minor and major subpopulation, and the minor and major subpopulations exchanged their roles between P24-A2 and P24-C, without evidence of recombination (**Fig. 5B**). Nonetheless, we identified multiple rare subpopulations at low frequencies carrying recombinant *gyrA* gene, indicating possible frequent recombination occurring at a low level.

The genome of *H. pylori* contains two copies of the *23S rRNA* gene, and the three isolates from patient P4 exhibited complex haplotypes of SNVs in the gene at subpopulation-levels. As shown in **Fig. 5C**, recombination occurred frequently within the *23S rRNA* gene, along with several likely *de novo* mutations. Among these subpopulations, GG and AG represent the two predominant genotypes at positions 2142 and 2143 of *23S rRNA*. However, because the two adjacent loci are expected to be linked, it remains unclear whether these genotypes arose primarily from recombination or *de novo* mutation. Nevertheless, the spread of the GG and AG genotypes across subpopulations is likely mediated by recombination.

Recombinant genomic regions had differential 5mC levels

We obtained genome-wide 5mC profiles (at isolate consensus level rather than the subpopulation level) of 39 *H. pylori* isolates from 14 patients by using nanopore sequencing (**Table S1**). Hierarchical clustering showed that most isolates clustered tightly by patients, except for those from P8 and P9-C (**Fig. S7**). Interestingly, we observed a significantly higher level of 5mC in coding regions compared to non-coding regions (two-sided Wilcoxon rank-sum test, $P < 0.001$; **Fig. 6A**).

The 5mC profiles from the same individuals were comparable. We found that the genomic regions with large differences in 5mC level coincided substantially with SNV clusters. Namely, the recombinant regions among the within-host subpopulations had distinguished epigenetics features. Moreover, the magnitude of difference in 5mc profiles corresponded to the subpopulation frequencies and their relationships. For example, the major subpopulation of P5-A1 differed from the dominant in P5-IA, and the recombinant region showed a large difference in 5mC levels (**Fig. 6B**). In P4, the largest 5mC difference was between P4-IA and P4-A2, whereas P4-A1 *versus* P4-A2 exhibited a smaller difference, consistent with their smaller difference in subpopulation frequencies (**Fig. 6C**). For isolates from P1, we extracted the genomic region around 1.4Mb that showed clear recombination among the subpopulations (**Fig. 4C**). The difference in 5mC corresponded to the spread of the recombinant regions among subpopulations; however, the small subpopulation frequency difference (<10%) between the P1-A1 and P1-A2 did not lead to a detectable difference in 5mC levels (**Fig. 6D** and **Fig. S8A**). The 5mC profile differences for the remaining isolates are provided in **Fig. S8B-F**.

Discussion

We characterized within-host subpopulations of *H. pylori* across multiple gastric regions. We observed the coexistence of two subpopulations within isolates from individual gastric biopsies, which was more common in the heteroresistance group than in the homoresistance group. Using a methodology integrating within-host diversity and haplotype analyses at the subpopulation level, we were able, for the first time, to resolve the relationships, relative abundances, and genetic material exchange among *H. pylori* subpopulations. Notably, recombination plays a critical role in the spread of mutations conferring resistance to LEV and CLA among subpopulations.

The methodology, not only captured within-host diversity across different gastric regions, but also depicted the diversity of *H. pylori* within the same gastric region, providing a clearer picture of how *H. pylori* colonizes gastric niches. The method relied on consensus-level SNPs could not detect the coexistence of multiple subpopulations and, in this study, largely underestimated within-host diversity, as shown in **Fig. 2B-F**. For example, the mixed infection of different strains in P22, as well as the coexistence of two subpopulations in many isolates, failed to be identified through a SNP-based analysis. On the other side, the methodology could recover the relative abundance of the subpopulations, and thus could assess the major and minor populations, which is informative for the microevolution analysis. In particular, we found that the coexistence of minor and major subpopulation in the gastric antrum tended to be more balanced, consistent with the model in which *H. pylori* diversification is initiated in the antrum and followed by dispersal to other gastric regions. The relative abundances are difficult to resolve by the labor-intensive single-colony genomic analysis.

Moreover, with a phased genomic variation analysis, we resolved the haplotypes at the subpopulation level, and observed extensive recomibiantion among sub-populations. Using this phasing workflow, the median chunk length was 2,583 bp, consistent with the kilobase-scale macro-import lengths (~1.3–3.8 kb) reported in recent whole-genome studies[37, 38]. It should be noted that our customized phasing workflow has limited sensitivity for micro-imports, as it uses a greedy implementation that tends to bridge gaps between adjacent imports and infer longer recombination chunks[39].

The haplotype analysis at the subpopulation level of the antibiotic resistance genes reveals how the mutations were harbored and spread within subpopulation in the gastric niche. In P9, a LEV resistance–associated *gyrA* haplotype dominated the isolate in IA, which was a recombinant between the minor and major haplotypes in A1. It might support a model in which the resistance mutation spreads via recombinant immigration from the antrum to other gastric regions, instead of immigration of minor and major subpopulation. In P24, the minor and major haplotypes in A2 and C are identical but with switch of roles; however, we observed extensive but low-frequency recombinant *gyrA* haplotypes. In P4, the two copies of *23S rRNA* exhibit a more complicated pattern of haplotypes. These findings imply that there might be a recombinant reservoir for resistant mutation variants.

Extending beyond genetic variation, we further explored whether epigenetic variation mirrors within-host population structure. Previous studies have reported the highly diverse methylomes of different strains of *H. pylori* [40-45]., caused by the 30–50 restriction–modification (RM) systems[41, 46] and on average ~19 DNA methyltransferases (MTases). However, there has no study of within-host diversity of *H. pylori* methylomes. In this study, we obtained and compared the genome-wide 5mC

profiles of 39 isolates from 14 patients. We found that 5mC differences between isolates within the same patient overlapped SNV-enriched regions, and the magnitude of these differences was related to the divergence of subpopulations. It provided the first evidence of how *H. pylori* subpopulations differed epigenetically, and the relatively large difference in recombination region suggest xenogeneic donors, possibly from other bacterial species.

This study has several limitations. First, the sample size is small, and patients were recruited from a single center, and there were no longitudinal samples for resolving the temporal dynamics of subpopulations. Second, although *H. pylori* positive isolates were subcultured once without further passaging, the culture might also alter the abundance of subpopulations. However, direct metagenomic sequencing of biopsies is unfeasible due to the low fraction of *H. pylori*. Third, our home-made phasing bioinformatic workflow highly depended on the SNV density and sequencing quality, such as PacBio HiFi read lengths, and the algorithm may merge adjacent import events, reducing detection sensitivity for short tracts (microimports). Finally, methylation profiling was obtained based on nanopore sequencing, and only the 5mC profiles were obtained. Larger sample size, multiple-center validation, and more experimental techniques are required to further address *H. pylori* within-host microevolution.

In summary, we provide a new framework for within-host diversity analysis of *H. pylori*, reveal the subpopulation structures of *H. pylori* within the gastric niche, and highlight the role of recombination in the diversification of *H. pylori* within hosts as well as the antibiotic-conferring mutation among the subpopulations.

Materials and methods

Isolate collection and antimicrobial susceptibility testing

This study was approved by the Medical Ethics Committee of Shenzhen Hospital of Southern Medical University (approval No. NYSZYYEC2024K125R001), and written informed consent was obtained from all participants. A total of 25 patients with chronic gastritis (6 females and 19 males) were enrolled in southern China. Gastric biopsies were obtained from two to four anatomical sites per patient, including the greater curvature of the antrum (A1), the lesser curvature of the antrum (A2), the incisura angularis (IA), and the gastric corpus (C). None of the participants had received antibiotic treatment within 1 month prior to sampling.

Helicobacter pylori was isolated and identified from biopsy specimens. Briefly, biopsies were homogenized, inoculated onto blood agar plates, and incubated in a tri-gas atmosphere (85% N_2, 10% CO_2, and 5% O_2) for 3–11 days. *H. pylori* colonies were confirmed by positive peroxidase, catalase, and oxidase reactions and by typical spiral morphology on microscopy. Positive isolates were subcultured once and stored as glycerol stocks at −80°C. Each isolate was labeled with patient and sampling-site information and was not passaged further.

Antimicrobial susceptibility to clarithromycin and levofloxacin was assessed using a growth/no-growth assay on blood agar. Bacterial suspensions were adjusted to a standardized turbidity and spread onto blood agar plates with or without antibiotic. Plates were incubated for 3 days under the same tri-gas conditions. Susceptibility was determined by comparing growth on antibiotic-containing plates with growth on antibiotic-free control plates.

Genome sequencing

Genomic DNA was extracted using the DNeasy Blood & Cell Kit (Qiagen, Hilden, Germany), dissolved in nuclease-free water, and incubated at 4°C overnight. DNA purity was evaluated using a NanoDrop spectrophotometer and a Qubit fluorometer, with A260/280 and A260/230 ratios used to assess protein and RNA contamination, respectively. DNA integrity was confirmed by agarose gel electrophoresis.

Short-read next-generation sequencing (NGS), Oxford Nanopore sequencing, and PacBio sequencing were performed. For NGS, libraries were prepared using the NEBNext DNA Library Prep Kit and sequenced on MGISEQ-2000 and Illumina NovaSeq 6000 platforms to generate 150-bp paired-end reads. For Oxford Nanopore sequencing, DNA ends were repaired using the NEBNext Ultra II End Repair Kit; barcodes were ligated using the NEB Blunt/TA Ligase Master Mix, followed by ligation of sequencing adapters. Sequencing was conducted on R9.4.1 flow cells using a MinION device (Oxford Nanopore Technologies). For PacBio HiFi sequencing, ~10-kb fragments were size-selected using BluePippin (Sage Science) and sequenced on a Sequel II system (PacBio, USA) in HiFi mode to generate circular consensus sequencing (CCS) reads.

Sequencing data quality control

Quality of NGS reads was assessed using FastQC (v0.11.8). Low-quality bases (Phred score < 30) at read termini were trimmed using Trimmomatic (v0.39). For Oxford Nanopore data, reads with a quality score < 7 were removed. Basecalling was performed with Guppy (v3.2.1), and reads were demultiplexed by barcode using Porechop with default settings. For PacBio HiFi data, CCS reads were generated from subreads using pbccs (v6.2.0) with parameters --chunk 1/5 --min-passed 0 -j 15, and

demultiplexing of barcoded CCS reads was performed using lima (v2.1).

Genome assembly

Assembly strategies were selected based on data availability. For isolates with both NGS and long-read data, genomes were first assembled de novo using long reads and then polished with NGS reads. For PacBio data, reads ≥1,000 bp were assembled using MECAT2 with an expected genome size of 1.6 Mbp. For Oxford Nanopore data, de novo assembly was performed using NECAT with an expected genome size of 1.6 Mbp and a minimum read length of 3,000 bp. Long-read assemblies were polished using Pilon (v1.23) with NGS reads. For circular genomes, the origin of replication was adjusted according to GC skew. Isolates with NGS data only were assembled using SPAdes.

Genome typing, annotation, and core genome inference

Isolate lineages were assigned using the *H. pylori* genotyping tool HPTT. Genome annotation was performed with Prokka (v1.13.6). The core genome was inferred using Roary (v3.11.2) based on annotated gene sets.

Intra-host SNP and subclonal iSNV detection

For each patient, the highest-quality assembly was selected as the patient-specific reference (PacBio assemblies were preferred when available). NGS reads from all isolates within the same patient were aligned to the reference using BWA (v0.7.17). Alignments were converted to mpileup format using SAMtools (v1.9). Variants were called using the iSNV-calling pipeline (v1.0) with the following filters: sequencing depth ≥50×, ≥5 reads supporting the variant allele, and mutant allele frequency (MuAF) >0.05. Variants were categorized as SNPs when MuAF ≥0.95 and as intra-host subclonal nucleotide variants (iSNVs) when 0.05 ≤ MuAF <0.95. For sensitivity

analyses in correlation testing with 5mC, alternative thresholds were applied (SNPs: MuAF ≥0.9 and ≥0.99; iSNVs: MuAF 0.1–0.9 and 0.2–0.8). Variants fixed at the same state across all isolates within a patient were excluded from intra-host SNP counts.

Phylogenetic analyses

A maximum-likelihood phylogeny was reconstructed from the core genome alignment using RAxML (v8.2.12) under the GTRGAMMAI model, with 1,000 rapid bootstrap replicates. To account for the impact of homologous recombination on tree topology, the tree was adjusted using ClonalFrameML (v1.12). The resulting phylogeny was visualized with iTOL (v6).

Subclonal phasing of genomic variation

To resolve within-stomach biogeography and subclonal structure, read-backed phasing was performed using long reads. Because colonies were isolated without additional passaging, subclonal heterogeneity present in the original biopsy was preserved. An anchor set of SNVs supported by both short reads and PacBio reads was defined. For each long read spanning at least two anchor loci, a local haplotype was assigned based on the alleles observed on that read.

Local haplotypes were extended along the genome in a greedy, genome-ordered procedure. The genome was scanned across anchor loci, and an interval was extended as long as each adjacent pair of loci within the interval was co-covered by at least one long read. When no read bridged the last two loci, the last locus initiated a new interval, yielding a series of phased blocks along the chromosome.

Within each phased block, reads sharing identical allele patterns were collapsed into haplotype groups. Relative abundance of each subclonal haplotype was estimated as the number of supporting reads normalized by total coverage in that block. Phased

haplotypes and their frequencies were used to infer subclonal composition across gastric regions and to delineate co-inherited variant clusters representing recombination blocks.

Phasing of drug-resistance loci

Phasing of resistance loci used the same read-backed strategy and was restricted to *23S rRNA* and *gyrA*. Short reads were aligned to locus-specific references, and pileups were generated with a minimum base quality of 20. Variants were called with depth ≥100, ≥5 reads supporting the nonreference allele, and MuAF ≥0.05. Samples with ≥5 variable sites per locus were retained for haplotype reconstruction.

Copy-resolved phasing of *23S rRNA* required unambiguous assignment of reads to the two genomic copies. Copy coordinates were obtained from genome annotations. Long reads were assigned to a specific copy only when they extended ≥400 bases beyond the homologous flanks in total. Reads assigned to each copy were trimmed to the *23S rRNA* interval and aligned using NGMLR. Haplotype sequences were reconstructed from short-read–defined variable sites, and only haplotypes supported by ≥10 long reads were retained.

For *gyrA*, the target interval was defined from annotations. Long reads mapping to this interval were extracted, realigned to a *gyrA* reference using NGMLR, and haplotypes were defined using the same criteria as for *23S rRNA*. For samples P9-A1 and P9-IA, Oxford Nanopore reads were used for phasing because PacBio data were unavailable. To mitigate single-base errors, preliminary haplotypes were clustered by sequence similarity; within each cluster, the most-supported haplotype was used as a centroid, and other haplotypes were corrected relative to the centroid to resolve likely sequencing errors and informative deletions. Corrected haplotypes were then

hierarchically clustered, and a consensus sequence was derived for each cluster to obtain the final copy-resolved haplotypes.

Methylation detection and analysis

Genomic DNA methylation was inferred from Oxford Nanopore fast5 files using Tombo (v1.5.1). Two reference strategies were applied: (i) the assembly of each isolate and (ii) the patient-specific reference genome. Methylation calls from these strategies were denoted M-1 and M-2, respectively.

The workflow comprised four steps: preprocessing to integrate basecalling information into fast5 files, resquiggling to align current signals to the reference genome, detection of modified bases, and export of methylation calls for downstream analyses. Tombo reports methylation calls separately for plus and minus strands; the strand matching the reference genome is designated as plus, with the complementary strand designated as minus.

Cytosines were considered 5mC-methylated when the Tombo dampened_fraction was ≥0.95 and coverage was ≥50. For whole-genome clustering of 5mC patterns, methylation data from M-1 were merged, and multiple-sequence alignment was performed across isolates from the same patient. Sites with methylation fraction ≥0.95 were coded as 1, and all other sites (including gaps) were coded as 0. A distance matrix was computed from the binary profiles using the fastdtw package in Python, followed by hierarchical clustering (complete linkage) using the linkage function in SciPy.

To compare recombination regions with differential 5mC, whole-genome alignments among intra-host isolates were generated using Mauve in Geneious Prime (v2022.2.1) to identify putative recombination tracts and to construct a patient-specific consensus sequence (CS). 5mC sites from each isolate were mapped onto the CS. To

reduce biases introduced by local sequence composition or structural variation, recombination regions containing large deletions or strongly asymmetric cytosine distributions were excluded. For each retained recombination region, a nearby non-recombining region of equal length was selected as a local control.

Statistical analysis

All statistical analyses were conducted in R (v4.1.0). Statistical significance was defined as $P \leq 0.05$ after adjustment where applicable. Continuous variables were compared using two-sided Wilcoxon rank-sum tests, and categorical variables were compared using two-sided Fisher's exact tests.

Conflict of interest

None declared.

Funding

This work is supported by National Natural Science Foundation of China (31870079).

Availability of Data and Materials

The whole-genome sequencing data generated in this study have been deposited in the Genome Sequence Archive (GSA) of the China National Center for Bioinformation (https://www.cncb.ac.cn/) under BioProject accession PRJCA041148.

Figure titles and legends

Fig. 1. Sampling strategy, resistance phenotypes, and phylogenetic relationships of 69 *Helicobacter pylori* isolates from 24 patients. (A) After isolation and culture of *H.*

pylori from gastric mucosal biopsy specimens, antimicrobial susceptibility testing was performed for two antibiotics, clarithromycin and levofloxacin. The heteroresistance group ($n = 14$) comprised patients whose isolates from different gastric regions showed discordant susceptibility to clarithromycin and/or levofloxacin. The homoresistance group ($n = 11$) comprised patients whose isolates from all sampled regions showed concordant susceptibility profiles. (B) A phylogenetic tree was constructed using the 69 isolates together with representative global *H. pylori* lineages. All isolates belonged to the hspEAsia lineage, except isolate P8-A1, which belonged to the hpAfrica1 lineage. (C) A core-genome phylogenetic tree of all isolates was rooted on *H. pylori* strain Puno135 (NC_017379.1). Branches in red indicate isolates from heteroresistant patients, whereas branches in blue indicate isolates from homoresistant patients. Patients are denoted by “P” followed by a number.

Fig. 2. Intra-host genetic diversity of 69 *H. pylori* isolates from 24 patients. (A) Schematic representation of two types of intra-host genomic variation in *H. pylori*. Using a patient-specific reference genome, intra-host single nucleotide polymorphisms (SNPs) were defined as fixed differences at a nucleotide site among isolates from the same patient (mutant allele frequency, MuAF $\geqslant 0.95$), where at least one isolate carried an alternative nucleotide at that site. Intra-host single nucleotide variants (iSNVs) were defined as polymorphic sites with $0.05 \leqslant$ MuAF < 0.95 among isolates from the same patient. (B) The number of SNPs per patient was determined using patient-specific reference genomes. Patients were categorized into heteroresistance (gray bars) and homoresistance groups (dark gray bars). A significant difference was observed between groups (two-sided Wilcoxon rank-sum test, $P < 0.001$). (C) The number of iSNVs per gastric region was determined using patient-specific reference genomes. For patients P8 and P22, striking within-patient differences in iSNV counts were observed between

gastric regions: one region harbored a very high number of iSNVs, whereas the other region harbored very few. (D) Isolate P8-A2 represented a nearly pure clone, whereas in the A1 region this clone accounted for only 18% of the population, with a distinct clone constituting the major population. (E) Isolate P22-A1 represented a pure clone, which also constituted the major clone (86%) in the corpus (C) region. (F) The number of iSNVs per isolate was determined using patient-specific reference genomes. Isolates were categorized into heteroresistance (gray bars) and homoresistance groups (dark gray bars). No significant difference was observed between groups (two-sided Wilcoxon rank-sum test, $P = 0.084$). Gastric regions are indicated as corpus (C), greater curvature of the antrum (A1), lesser curvature of the antrum (A2), and incisura angularis (IA).

Fig. 3. Subpopulation structure of *H. pylori* across gastric regions in patients with abundant intra-host variants. (A–F) Distributions of mutant allele frequencies (MuAFs) of SNVs in isolates from six selected patients (P4, P5, P7, P12, P23, and P24; isolates with ⩾ 500 SNVs). These MuAF distributions are approximately unimodal, indicating that each sequenced colony is dominated by a single major clone. Patients P4, P5, P7, and P12 belong to the heteroresistance group, whereas P23 and P24 belong to the homoresistance group. Each histogram corresponds to one isolate from a specific gastric region. Colors indicate regional distribution patterns of SNVs, distinguishing variants confined to a single region (blue) from those shared between two (green) or three (orange) regions. (G) Inferred subpopulation composition for each gastric region in the six patients is summarized as pie charts; numbers in parentheses indicate the total number of SNV sites for each patient–region combination. Darker blue sectors represent subpopulations carrying a larger number of distinct variants (greater divergence), whereas lighter blue sectors represent subpopulations more similar to the

dominant clone. (H) Minor-subpopulation fractions were significantly higher in the antrum than in the incisura angularis or corpus (two-sided Wilcoxon rank-sum test, P = 0.011).

Fig. 4. Subpopulation structure and clustered iSNVs ("SNV chunks") in *H. pylori* revealed by phasing. (A) MuAF distributions of SNVs in four gastric regions from patient P1, showing a bimodal pattern. (B) Genomic distribution of SNVs in P1, calculated in 1-kb windows with a 1-bp step. Peaks indicate SNV-rich segments, and grey shaded regions mark five SNV-dense blocks selected for phasing analysis. (C) Phasing results for P1. Pie charts indicate the relative abundance of major and minor clones, and horizontal bars show contiguous SNV-dense segments ("SNV chunks") along the genome. Orange segments denote SNV chunks shared by three regions, whereas blue segments denote chunks unique to a single region. (D) MuAF distributions of SNVs in two gastric regions from patient P6, also displaying a multimodal pattern. (E) Genomic distribution of SNVs in P6, calculated in 1-kb windows with a 1-bp step. Peaks indicate SNV-rich segments, and grey shaded regions mark five SNV-dense blocks selected for phasing analysis. (F) Phasing results for P6. Pie charts indicate the proportions of major and minor clones, and horizontal bars show SNV chunks; blue segments represent region-specific chunks, and green segments represent chunks shared between the two regions. (G) Length distribution of SNV chunks on a logarithmic scale, with most chunks spanning a few kilobases (around 3 kb).

Fig. 5. Phased haplotypes at levofloxacin- and clarithromycin-resistance loci reveal complex within-host subpopulation structure of *H. pylori*. (A–B) Phased SNV chunks at resistance loci in representative patients. For each locus, the top track shows the corresponding region of the Puno135 reference gene (NC_017379.1), with

short blue ticks marking the positions of phased iSNVs. The dendrogram on the left summarizes relationships among phased haplotypes, and the central panel depicts contiguous phased SNV chunks along the locus. Resistance-associated mutations are indicated by red arrowheads. Colored bars on the right show the relative abundance of each haplotype across gastric regions. (A) Phased haplotypes of an SNV chunk spanning the *gyrA* locus in patient P9, including the resistance-associated substitution G271A (D91N). (B) Phased haplotypes of the *gyrA* locus in patient P24, showing multiple low-frequency haplotypes derived from combinations of variants present in the major and minor clones. (C) Phased haplotypes of an SNV chunk spanning the *23S rRNA* gene in patient P4, including the resistance-associated substitutions A2142G and A2143G. Both the SNV chunk and additional point mutations (green arrowheads) within this region contribute to the coexistence of multiple haplotypes.

Figure 6. Recombinant genomic regions show differential 5mC levels. (A) The fraction of 5mC sites was significantly higher in coding regions than in noncoding regions (two-sided Wilcoxon rank-sum test, $P < 0.001$). (B) In P5, the 5-kb genomic window with the highest SNV density. Bottom left, the corresponding profile of differential 5mC levels between isolates, showing concordance with the SNV-enriched region. Right, inferred subpopulation composition across gastric regions, indicating larger differences in subpopulation proportions between regions compared in the methylation panel. (C) In P4, the top left panel shows the 5-kb window with the highest SNV density, the bottom left panel shows differential 5mC levels, and the right panel shows inferred subpopulation composition across gastric regions. (D) In P1, the top left panel shows the 5-kb window with the recombination chunks region was illustrated in Fig. 4C, the bottom left panel shows differential 5mC levels, and the right panel shows inferred subpopulation composition across gastric regions.

References

1. Malfertheiner, P., et al., *Management of Helicobacter pylori infection: the Maastricht VI/Florence consensus report.* Gut, 2022.
2. Jing, Z.W., et al., *Design and evaluation of novel pH-sensitive ureido-conjugated chitosan/TPP nanoparticles targeted to Helicobacter pylori.* Biomaterials, 2016. **84**: p. 276-285.
3. Chen, Y.C., et al., *Global Prevalence of Helicobacter pylori Infection and Incidence of Gastric Cancer Between 1980 and 2022.* Gastroenterology, 2024. **166**(4): p. 605-619.
4. Li, Y., et al., *Global prevalence of Helicobacter pylori infection between 1980 and 2022: a systematic review and meta-analysis.* Lancet Gastroenterol Hepatol, 2023. **8**(6): p. 553-564.
5. Uemura, N., et al., *Helicobacter pylori infection and the development of gastric cancer.* N Engl J Med, 2001. **345**(11): p. 784-9.
6. Kusters, J.G., A.H. van Vliet, and E.J. Kuipers, *Pathogenesis of Helicobacter pylori infection.* Clin Microbiol Rev, 2006. **19**(3): p. 449-90.
7. Fukase, K., et al., *Effect of eradication of Helicobacter pylori on incidence of metachronous gastric carcinoma after endoscopic resection of early gastric cancer: an open-label, randomised controlled trial.* The Lancet, 2008. **372**(9636): p. 392-397.
8. Rawson, T.M., et al., *A systematic review of clinical decision support systems for antimicrobial management: are we failing to investigate these interventions appropriately?* Clin Microbiol Infect, 2017. **23**(8): p. 524-532.
9. Thrift, A.P., T.N. Wenker, and H.B. El-Serag, *Global burden of gastric cancer: epidemiological trends, risk factors, screening and prevention.* Nat Rev Clin Oncol, 2023. **20**(5): p. 338-349.
10. Ford, A.C., Y. Yuan, and P. Moayyedi, *Helicobacter pylori eradication therapy to prevent gastric cancer: systematic review and meta-analysis.* Gut, 2020. **69**(12): p. 2113-2121.
11. Choi, I.J., et al., *Helicobacter pylori Therapy for the Prevention of Metachronous Gastric Cancer.* N Engl J Med, 2018. **378**(12): p. 1085-1095.
12. Savoldi, A., et al., *Prevalence of Antibiotic Resistance in Helicobacter pylori: A Systematic Review and Meta-analysis in World Health Organization Regions.* Gastroenterology, 2018. **155**(5): p. 1372-1382.e17.

13. Ng, H.Y., W.K. Leung, and K.S. Cheung, *Antibiotic Resistance, Susceptibility Testing and Stewardship in Helicobacter pylori Infection.* Int J Mol Sci, 2023. **24**(14).

14. Dubnau, D. and M. Blokesch, *Mechanisms of DNA Uptake by Naturally Competent Bacteria.* Annu Rev Genet, 2019. **53**: p. 217-237.

15. Stingl, K., et al., *Composite system mediates two-step DNA uptake into Helicobacter pylori.* Proc Natl Acad Sci U S A, 2010. **107**(3): p. 1184-9.

16. Ailloud, F., I. Estibariz, and S. Suerbaum, *Evolved to vary: genome and epigenome variation in the human pathogen Helicobacter pylori.* FEMS Microbiol Rev, 2021. **45**(1).

17. Suerbaum, S., et al., *Free recombination within Helicobacter pylori.* Proc Natl Acad Sci U S A, 1998. **95**(21): p. 12619-24.

18. Suerbaum, S. and C. Josenhans, *Helicobacter pylori evolution and phenotypic diversification in a changing host.* Nat Rev Microbiol, 2007. **5**(6): p. 441-52.

19. Lin, Z., M. Nei, and H. Ma, *The origins and early evolution of DNA mismatch repair genes--multiple horizontal gene transfers and co-evolution.* Nucleic Acids Res, 2007. **35**(22): p. 7591-603.

20. Linz, B., et al., *A mutation burst during the acute phase of Helicobacter pylori infection in humans and rhesus macaques.* Nat Commun, 2014. **5**: p. 4165.

21. Suerbaum, S. and F. Ailloud, *Genome and population dynamics during chronic infection with Helicobacter pylori.* Curr Opin Immunol, 2023. **82**: p. 102304.

22. Björkholm, B., et al., *Mutation frequency and biological cost of antibiotic resistance in Helicobacter pylori.* Proc Natl Acad Sci U S A, 2001. **98**(25): p. 14607-12.

23. Kang, J.M., N.M. Iovine, and M.J. Blaser, *A paradigm for direct stress-induced mutation in prokaryotes.* Faseb j, 2006. **20**(14): p. 2476-85.

24. Luo, Q., et al., *Clinical relevance, mechanisms, and evolution of polymyxin B heteroresistance carbapenem-resistant Klebsiella pneumoniae: A genomic, retrospective cohort study.* Clin Microbiol Infect, 2024. **30**(4): p. 507-514.

25. Draper, J.L., et al., *Fallacy of the Unique Genome: Sequence Diversity within Single Helicobacter pylori Strains.* mBio, 2017. **8**(1).

26. Mi, M., et al., *Heterogeneity of Helicobacter pylori Strains Isolated from Patients with Gastric Disorders in Guiyang, China.* Infect Drug Resist, 2021. **14**: p. 535-545.

27. Israel, D.A., et al., *Helicobacter pylori genetic diversity within the gastric niche of a single human host.* Proc Natl Acad Sci U S A, 2001. **98**(25): p. 14625-30.

28. Jackson, L.K., et al., *Helicobacter pylori diversification during chronic infection within a single host generates sub-populations with distinct phenotypes.* PLoS Pathog, 2020. **16**(12): p. e1008686.

29. Ailloud, F., et al., *Within-host evolution of Helicobacter pylori shaped by niche-specific adaptation, intragastric migrations and selective sweeps.* Nat Commun, 2019. **10**(1): p. 2273.

30. Patterson, M., et al., *WhatsHap: Weighted Haplotype Assembly for Future-Generation Sequencing Reads.* J Comput Biol, 2015. **22**(6): p. 498-509.

31. Edge, P., V. Bafna, and V. Bansal, *HapCUT2: robust and accurate haplotype assembly for diverse sequencing technologies.* Genome Res, 2017. **27**(5): p. 801-812.

32. Chin, C.S., et al., *Phased diploid genome assembly with single-molecule real-time sequencing.* Nat Methods, 2016. **13**(12): p. 1050-1054.

33. Cheng, H., et al., *Haplotype-resolved de novo assembly using phased assembly graphs with hifiasm.* Nat Methods, 2021. **18**(2): p. 170-175.

34. Garg, S., et al., *Chromosome-scale, haplotype-resolved assembly of human genomes.* Nat Biotechnol, 2021. **39**(3): p. 309-312.

35. Didelot, X. and D.J. Wilson, *ClonalFrameML: efficient inference of recombination in whole bacterial genomes.* PLoS Comput Biol, 2015. **11**(2): p. e1004041.

36. Cao, Q., et al., *Progressive genomic convergence of two Helicobacter pylori strains during mixed infection of a patient with chronic gastritis.* Gut, 2015. **64**(4): p. 554-61.

37. Bubendorfer, S., et al., *Genome-wide analysis of chromosomal import patterns after natural transformation of Helicobacter pylori.* Nat Commun, 2016. **7**: p. 11995.

38. Kulick, S., et al., *Mosaic DNA imports with interspersions of recipient sequence after natural transformation of Helicobacter pylori.* PLoS One, 2008. **3**(11): p. e3797.

39. Lin, E.A., et al., *Natural Transformation of Helicobacter pylori Involves the Integration of Short DNA Fragments Interrupted by Gaps of Variable Size.* PLOS Pathogens, 2009. **5**(3): p. e1000337.

40. Wilkinson, D.J., et al., *Genomic diversity of Helicobacter pylori populations from different regions of the human stomach.* Gut Microbes, 2022. **14**(1): p. 2152306.

41. Vasu, K. and V. Nagaraja, *Diverse functions of restriction-modification systems in addition to cellular defense.* Microbiol Mol Biol Rev, 2013. **77**(1): p. 53-72.
42. Alm, R.A., et al., *Genomic-sequence comparison of two unrelated isolates of the human gastric pathogen Helicobacter pylori.* Nature, 1999. **397**(6715): p. 176-80.
43. Krebes, J., et al., *The complex methylome of the human gastric pathogen Helicobacter pylori.* Nucleic Acids Res, 2014. **42**(4): p. 2415-32.
44. Nell, S., et al., *Genome and Methylome Variation in Helicobacter pylori With a cag Pathogenicity Island During Early Stages of Human Infection.* Gastroenterology, 2018. **154**(3): p. 612-623.e7.
45. Estibariz, I., et al., *The core genome m5C methyltransferase JHP1050 (M.Hpy99III) plays an important role in orchestrating gene expression in Helicobacter pylori.* Nucleic Acids Res, 2019. **47**(5): p. 2336-2348.
46. Yano, H., et al., *Networking and Specificity-Changing DNA Methyltransferases in Helicobacter pylori.* Front Microbiol, 2020. **11**: p. 1628.

Figure 1

Click here to access/download;Figure;Figure 1.pdf

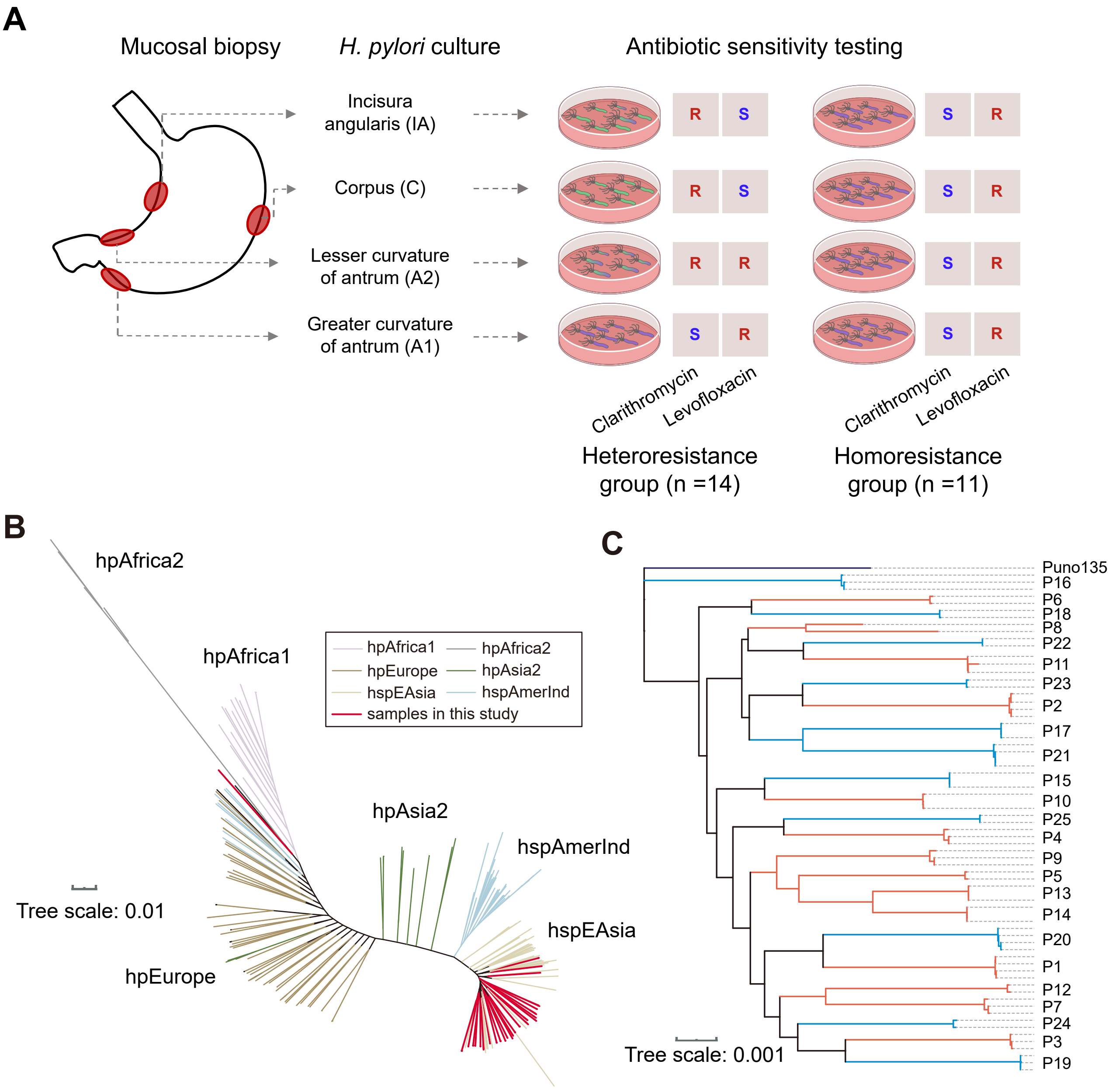

Figure 2

Click here to access/download;Figure;Figure 2.pdf

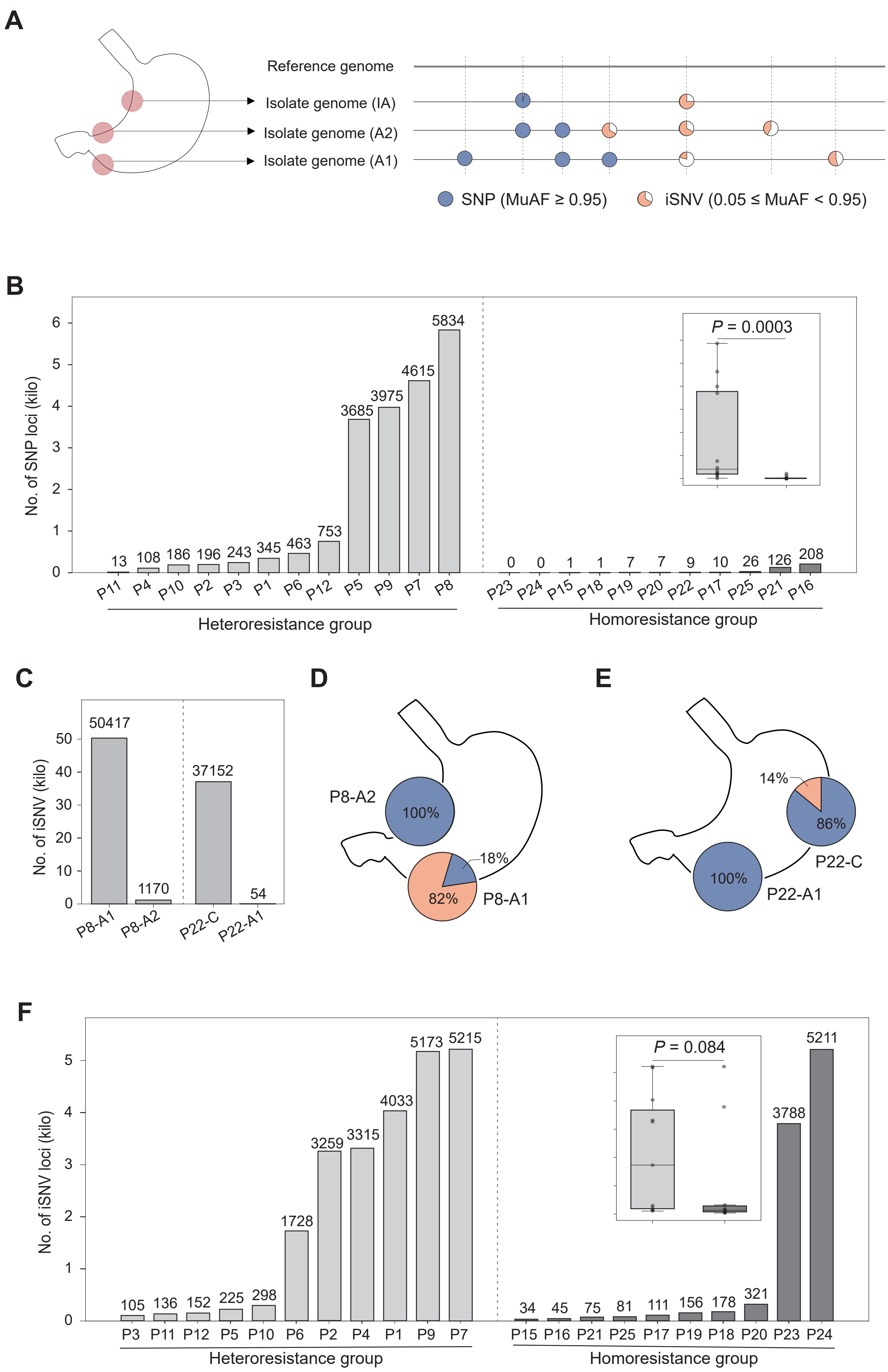

Figure 3

Click here to access/download;Figure;Figure 3.pdf

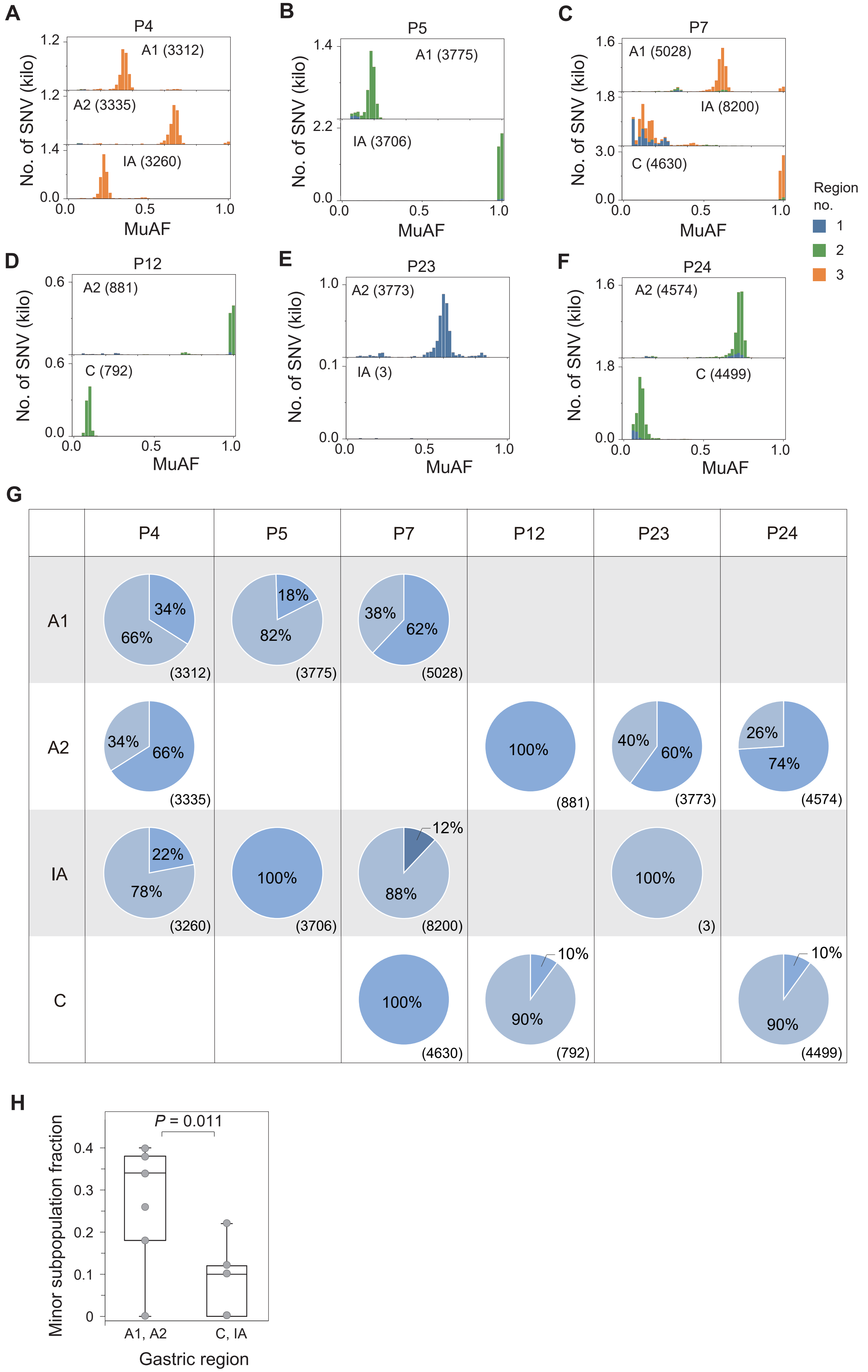

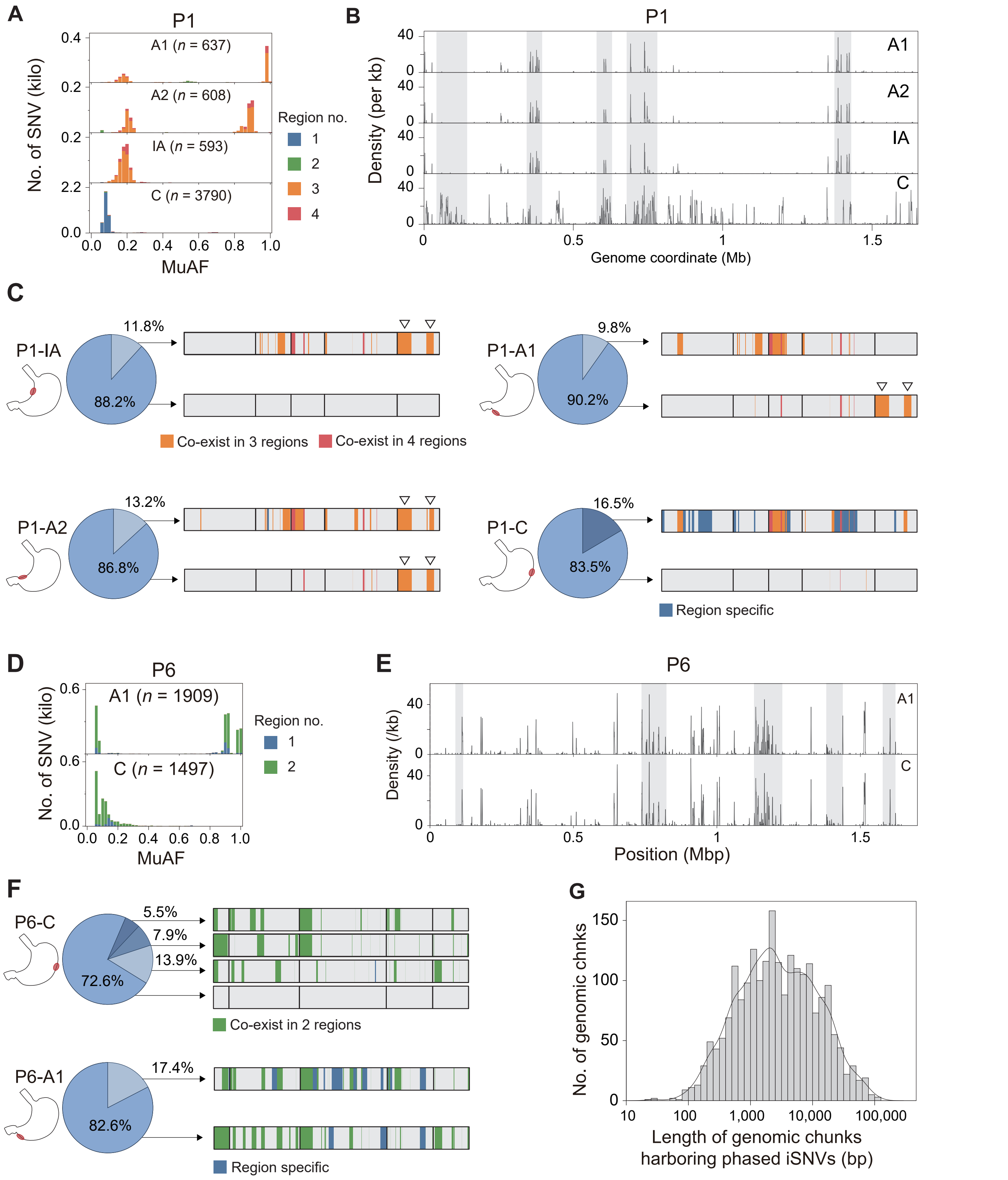
A
P1
A1 (n = 637)
A2 (n = 608)
IA (n = 593)
C (n = 3790)
No. of SNV (kilo)
MuAF
Region no.
1
2
3
4
B
P1
Density (per kb)
Genome coordinate (Mb)
A1
A2
IA
C
C
P1-IA
11.8%
88.2%
P1-A1
9.8%
90.2%
Co-exist in 3 regions
Co-exist in 4 regions
P1-A2
13.2%
86.8%
P1-C
16.5%
83.5%
Region specific
D
P6
A1 (n = 1909)
C (n = 1497)
No. of SNV (kilo)
MuAF
Region no.
1
2
E
P6
Density (/kb)
Position (Mbp)
A1
C
F
P6-C
5.5%
7.9%
13.9%
72.6%
Co-exist in 2 regions
P6-A1
17.4%
82.6%
Region specific
G
No. of genomic chnks
Length of genomic chunks
harboring phased iSNVs (bp)

Figure 5
Click here to access/download;Figure;Figure 5.pdf

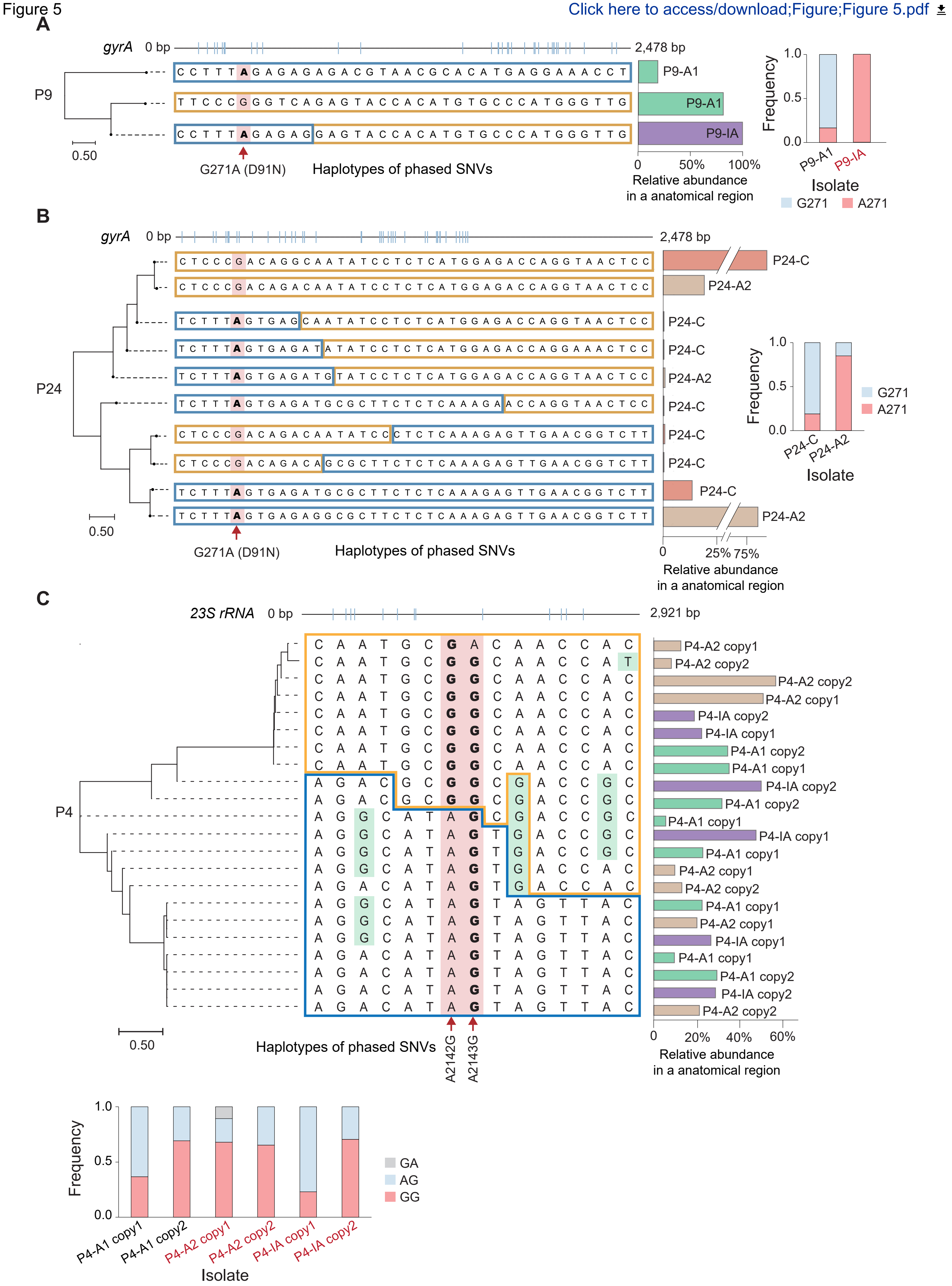

Figure 6 Click here to access/download;Figure;Figure 6.pdf

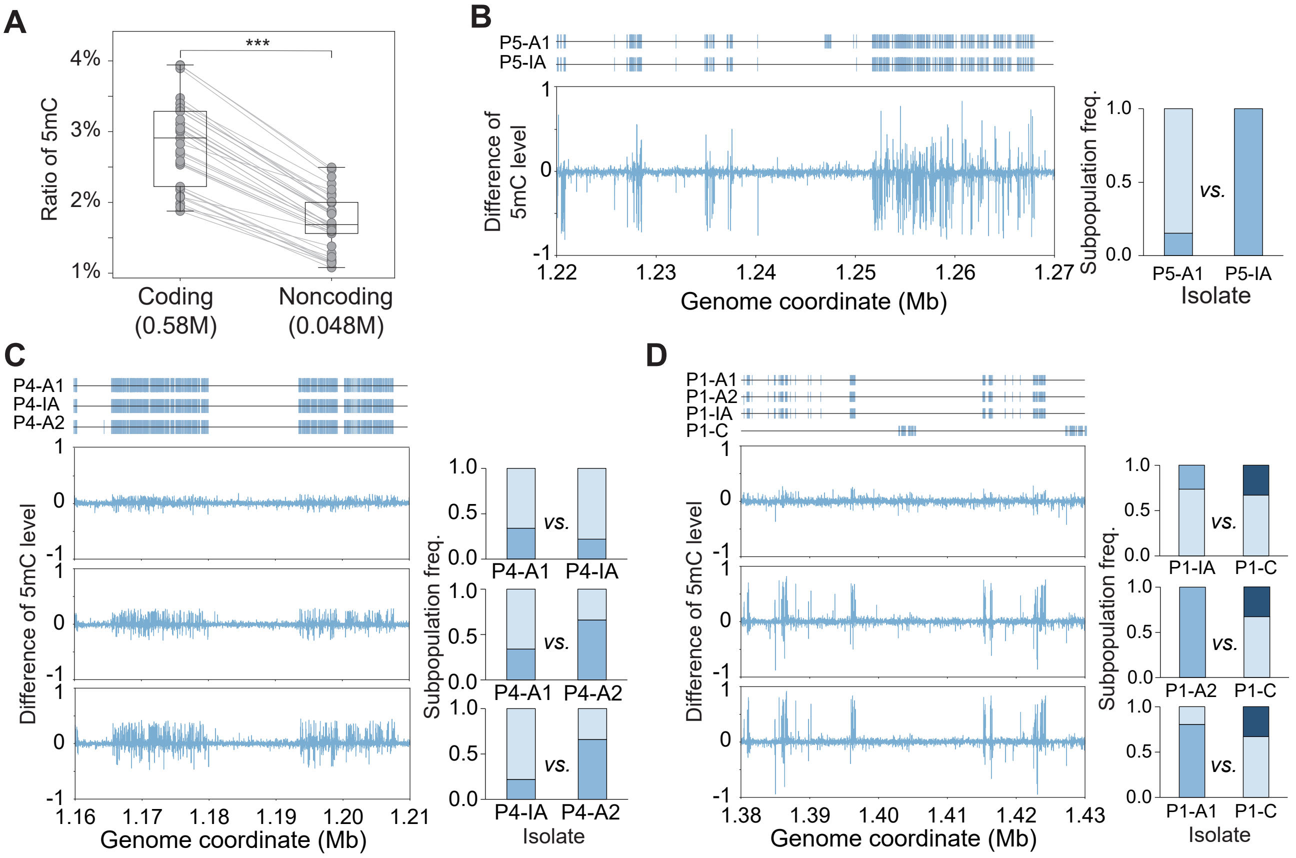

Supplementary Figure

Click here to access/download
Supplementary Material
supplementary figure.rar

Supplementary Table

Click here to access/download
Supplementary Material
supplementary table.xlsx

Graphical Abstract

Click here to access/download;Graphical Abstract;Graphical Abstract.pdf

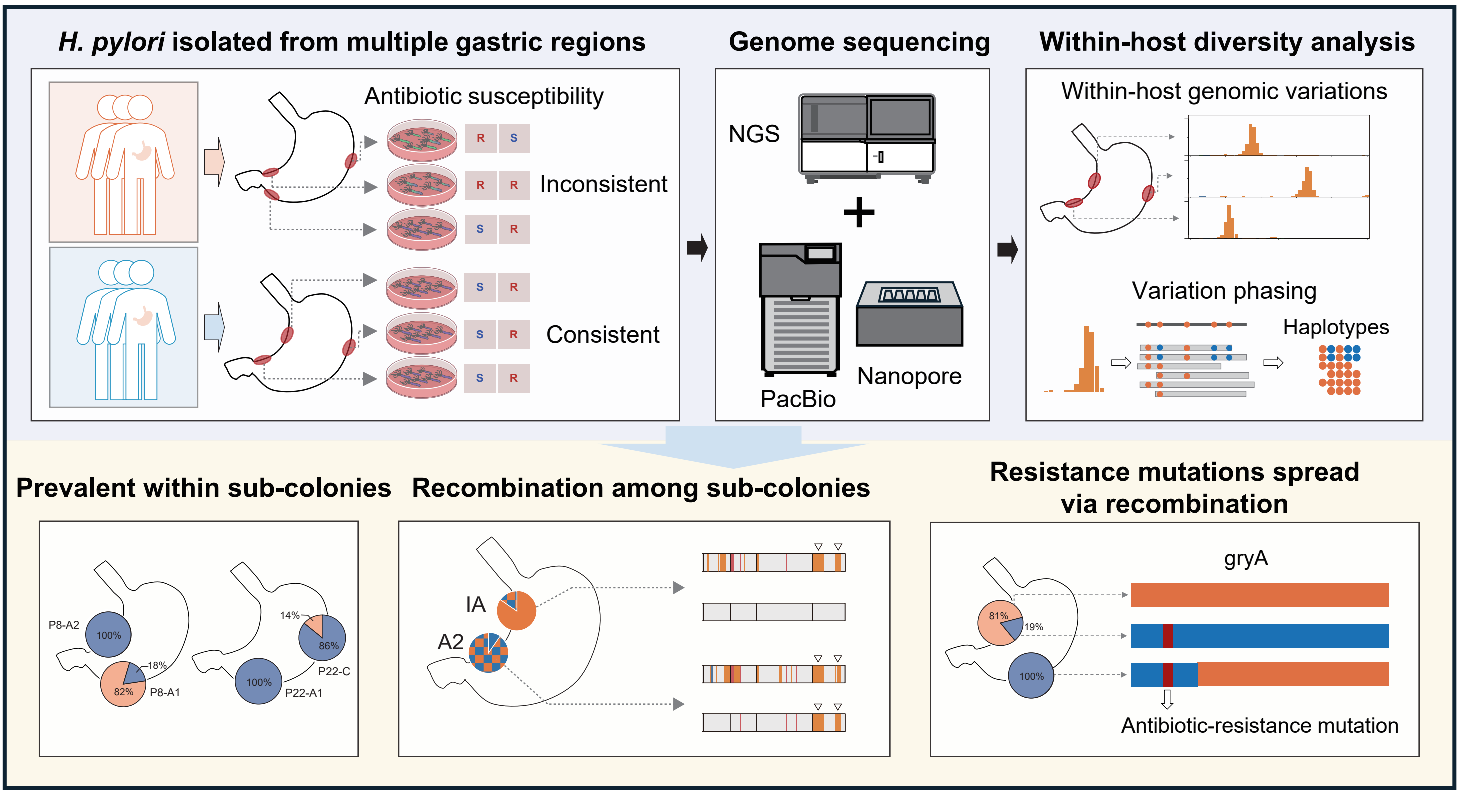

Graphical Abstract Text

Click here to access/download;Graphical Abstract;Graphical Abstract text.docx

Helicobacter pylori was isolated from multiple gastric regions of chronic gastritis patients with diverse antibiotic susceptibility. Within-host diversity and phased variant analysis reveal the structure of *Helicobacter pylori* subpopulations in the stomach and frequent recombination, which can cause the spread of antibiotic resistance mutations.
