# Supplementary material for: Within-host diversity and phased variant analysis reveal structures and recombination of Helicobacter pylori subpopulations in stomach
Source: Gigascience. 2026 Apr 16;15:giag046. doi: 10.1093/gigascience/giag046 (PMC13188224; doi:10.1093/gigascience/giag046)
Supplement: giag046_Authors_Response_To_Reviewer_Comments_original_submission [file giag046_authors_response_to_reviewer_comments_original_submission.pdf]

Author's Response To Reviewer Comments

Dear Dr. Dongni Ma,

Thank you for handling our manuscript, “Within-host diversity and phased variant analysis reveal structures and recombination of Helicobacter pylori subpopulations in stomach” (GIGA-D-26-00029), and for giving us the opportunity to revise it. We appreciate the careful review and the helpful comments from you and the reviewers, which have improved the clarity and quality of the manuscript. We have addressed the points raised and revised the manuscript accordingly. Our point-by-point responses to the reviewers' comments are provided below, and all changes in revised version of the manuscript have been marked using Track Changes.
Thank you for your helpful suggestion. We have now registered our software resource in the relevant databases and included the corresponding identifiers in the revised manuscript. Specifically, the software has been registered in bio.tools (bio.tools ID: hp-phasing; URL: https://bio.tools/hp-phasing) and SciCrunch (RRID: SCR_028195; URL: https://rrid.site/data/record/nlx_144509-1/SCR_028195/resolver?q=HP-Phasing&l=HP-Phasing&i=rrid:scr_028195). In addition, we have registered the computational workflow in WorkflowHub (URL: https://workflowhub.eu/projects/439) and cited the corresponding record in the relevant sections of the revised manuscript.

Thank you for your time and consideration.

Best regards,
Ming Ni
Academy of Military Medical Sciences

Response to reviewers：

Reviewer 1

Comment 1
Subculturing Effects: In the Methods, the authors mention that isolates were subcultured once. Although they state this was done to preserve subclonal heterogeneity, any culture step may impose a selective bottleneck. It would be helpful to include a brief sentence addressing the potential risk of losing low-frequency subpopulations during this single passage.
Response:
We agree and thank the reviewer for this important comment. Even a single subculture step may introduce selective pressure and therefore carries a risk of losing low-frequency subpopulations. In this study, the isolates were subcultured only once in an effort to minimize culture-induced changes and preserve the original population characteristics as much as possible. At present, direct characterization from biopsy specimens is not readily feasible for this purpose, because sequencing of biopsy material would be overwhelmingly dominated by host DNA, given the large disparity in genome size between the human genome (~3 Gb) and the H. pylori genome (~1.6 Mb). In addition, owing to the extremely high genetic diversity of H. pylori, targeted capture approaches based on probes or primers are not well suited for comprehensive characterization of within-sample subpopulations. Therefore, we consider analysis based on the initially culture-positive bacterial population, with only a single passage, to be the most feasible and appropriate approach currently available. We nevertheless agree that this single passage may still result in the loss of low-frequency subpopulations. We have added this clarification to both the Methods and Discussion sections of the revised manuscript (Lines 404 and 370–377).

Comment 2
The study focuses on 5mC. Given that 6mA is also highly prevalent in H. pylori and often associated with restriction-modification (R-M) systems, a brief comment in the Discussion on why 6mA was not analyzed (or its potential role in future studies) would strengthen the epigenetic section.
Response:

We thank the reviewer for this helpful suggestion. We agree that 6mA may also play an important role in H. pylori, particularly in relation to restriction–modification systems. In the present study, we did not analyze 6mA because no official Dorado model for 6mA calling was available for the Nanopore R9.4.1 data used in our workflow. In addition, compared with newer ONT chemistries, R9.4.1 generally provides lower accuracy for methylation detection and is more susceptible to signal noise, which further limited a reliable genome-wide analysis of 6mA in this dataset. We have now added a brief comment in the Discussion to clarify this point and to highlight the potential importance of 6mA for future studies using updated sequencing chemistries and basecalling models (Lines 357–361).

Reviewer 2

Major Comment 1
The sharing of iSNVs is presented in a pie chart, which reveals the subpopulations of H. pylori across different gastric regions. This is one of the main findings of the study. However, the authors did not show the sharing of SNPs between or among different gastric regions. Since this is a conventional and widely used method, analysis based on SNPs should be included.
Response:
Thank you for this helpful suggestion. We agree that a conventional SNP-based analysis should be included to complement the iSNV-based results. We have therefore added a brief description of the SNP-sharing analysis in the Results and provided Venn diagrams in the Supplementary Materials. This analysis showed heterogeneous overlap patterns across patients, and shared SNPs involving IA or C usually also included A1 and/or A2, whereas direct overlap between IA and C was uncommon (Lines 201–204; Fig. S3).

Major Comment 2
There is apparent discrepancy between the resistance-associated mutations and the antimicrobial susceptibility results. For example, in P24-A2, the subpopulation carrying the resistance-associated gyrA mutation appears to be the major one, whereas the isolate is reported as susceptible to levofloxacin. An explanation of this inconsistency would strengthen the interpretation of the result.
Response:
Thank you for pointing this out. We agree that this apparent inconsistency between the resistance-associated gyrA mutation and the susceptible levofloxacin phenotype in P24-A2 needs clarification. One possible explanation is that the antimicrobial susceptibility test was based on isolate picking and culture, which may not fully preserve the original subpopulation composition in the biopsy. When multiple subpopulations coexist, the population represented in the phenotypic assay may therefore differ from the relative abundance inferred from sequencing. This is a general limitation of culture-based susceptibility testing in the setting of mixed subpopulations. We have added a brief discussion to clarify this point in the revised manuscript (Lines 335–342).

Major Comment 3
The authors should add discussion about the clinical significance of these findings for H. pylori treatment.
Response:
We thank the reviewer for this helpful comment. We have added a brief paragraph in the Discussion to address the potential clinical significance of our findings for H. pylori treatment. In particular, we note that within-host heterogeneity of resistance-associated subpopulations may limit the representativeness of single-isolate or single-colony testing, and that analysis of multiple colonies and, where feasible, sampling from more than one gastric site may help provide a more complete assessment of resistance-associated variation, especially in cases of treatment failure or suspected mixed populations (Lines 343–351).

Minor Comment 1
Table 1 needs clearer annotation and more consistent anatomical labeling. In lines 105–108, the legend explains only R and S, but the meaning of "-" is not defined. In addition, the description of A1/A2 in lines 106–107 does not appear to be fully consistent with the terminology used elsewhere in the manuscript.
Response:
Thank you for pointing this out. We have revised the annotation of Table 1 to define the meaning of "–" and to make the anatomical labeling consistent with the terminology used in the main text. Specifically, A1 and A2 are now defined as the greater curvature of the antrum and the lesser curvature of the antrum,

respectively (Lines 108–111).

Minor Comment 2
There are a few singular/plural agreement issues. Line 142: “H. pylori isolate from the heteroresistance group” and line 305: “coexistence of minor and major subpopulation” likely should be plural.
Response:
Thank you for pointing this out. We have corrected these singular/plural agreement issues throughout the revised manuscript, including the examples noted here (Lines 145 and 313).

Minor Comment 3
Methodological descriptions could be slightly clearer. In lines 411–412, the statement “the origin of replication was adjusted according to GC skew” would benefit from a brief indication of the software or procedure used.
Response:
Thank you for this helpful suggestion. We agree that this methodological description should be clearer. We have revised the Methods section to briefly indicate the procedure used for GC skew-based adjustment of the genomic start position. Specifically, GC skew was calculated as (G−C)/(G+C) in 100-bp windows across the genome, the cumulative GC skew was then obtained, and the position with the minimum cumulative GC skew was used as the new sequence start. Sequences were reverse-complemented when necessary to ensure a consistent orientation (Lines 443–447).

Minor Comment 4
The presentation of resistance genotype and phenotype could be made more explicit. For example, the correspondence between resistance-associated mutations, phased subpopulations, and antimicrobial susceptibility results is not always easy to follow directly from the figures and text. It would be helpful to make these relationships more explicit, especially in the cases discussed around lines 239–262.
Response:
Thank you for this helpful suggestion. We agree that these relationships were not always easy to follow directly from the original text. We have revised the Results section to describe more explicitly the correspondence between resistance-associated mutations, phased subpopulations, and antimicrobial susceptibility phenotypes in the representative cases discussed in this section (Lines 251–262).

Minor Comment 5
The terminology for iSNVs should be used more consistently in the Results and figure legends. For example, lines 137–138 refer to “intra-region single nucleotide variations (iSNVs)”, whereas lines 536–537 use “intra-host single nucleotide variants (iSNVs)”. These should be standardized.
Response:
Thank you for pointing this out. We agree that the terminology for iSNVs should be used more consistently. We have revised the manuscript and figure legends to standardize the term as “intra-host single nucleotide variants (iSNVs)” throughout the text, and we now use more specific wording only when referring to iSNVs within a particular gastric region (Line 141).

em Editorial Manager®

Cookie Preference Center

We use cookies which are necessary to make our site work. We may also use additional cookies to analyze, improve and personalize our content and your digital experience. For more information, see our Cookie Policy.

You may choose not to allow some types of cookies. However, blocking some types may impact your experience of our site and the services we are able to offer. See the different category headings below to find out more or change your settings.

Allow all

Manage Consent Preferences

Strictly Necessary Cookies

Always active

These cookies are necessary for the website to function and cannot be switched off in our systems. They are usually only set in response to actions made by you which amount to a request for services, such as setting your privacy preferences, logging in or filling in forms. You can set your browser to block or alert you about these cookies, but some parts of the site will not then work.

Functional Cookies

These cookies enable the website to provide enhanced functionality and personalisation. They may be set by us or by third party providers whose services we have added to our pages. If you do not allow these cookies then some or all of these services may not function properly.

Performance Cookies

These cookies allow us to count visits and traffic sources so we can measure and improve the performance of our site. They help us to know which pages are the most and least popular and see how visitors move around the site.

Targeting Cookies

These cookies may be set through our site by our advertising partners. They may be used by those companies to build a profile of your interests and show you relevant adverts on other sites. If you do not allow these cookies, you will experience less targeted advertising.

Cookie List

Clear

Apply Cancel
Consent Leg.Interest

Confirm my choices

Power
