# Supplementary material for: Within-host diversity and phased variant analysis reveal structures and recombination of Helicobacter pylori subpopulations in stomach
Source: Gigascience. 2026 Apr 16;15:giag046. doi: 10.1093/gigascience/giag046 (PMC13188224; doi:10.1093/gigascience/giag046)
Supplement: giag046_Reviewer_1_Report_original_submission — Reviewer 1 -- 3/16/2026 [file giag046_reviewer_1_report_original_submission.pdf]

Reviewer Report

Title: Within-host diversity and phased variant analysis reveal structures and recombination of Helicobacter pylori subpopulations in stomach

Version: Original Submission **Date:** 3/16/2026

Reviewer name: Reviewer 1

Reviewer Comments to Author:

This manuscript provides a high-quality, comprehensive investigation into the intra-host genomic diversity and microevolution of Helicobacter pylori. By utilizing a combination of ultra-deep short-read and long-read (PacBio HiFi/Nanopore) sequencing, the authors successfully challenge the traditional "single-biopsy, single-strain" paradigm.The most significant contribution of this work is the development of a custom phasing bioinformatics workflow that allows for the resolution of subpopulation-level genomic regions. This technical advancement enables a direct observation of how homologous recombination drives the spread of antibiotic resistance (specifically for levofloxacin and clarithromycin) across different gastric niches. The integration of 5mC methylome analysis adds an extra layer of depth, revealing that recombinant regions often harbor distinct epigenetic signatures. This study is timely and provides crucial insights into the mechanisms of "heteroresistance," which has profound implications for clinical eradication failures.Key Strengths:1. Methodological Innovation: The use of phased variant analysis to reconstruct subpopulation structures is a significant improvement over standard SNP-based methods, which often underestimate within-host diversity.2. Clinical Relevance: The study directly addresses why antibiotic susceptibility can vary across different regions of the same stomach, offering a genomic explanation for heteroresistance.Suggestions for Improvement:1. Subculturing Effects: In the Methods, the authors mention that isolates were subcultured once. Although they state this was done to preserve subclonal heterogeneity, any culture step may impose a selective bottleneck. It would be helpful to include a brief sentence addressing the potential risk of losing low-frequency subpopulations during this single passage.2. Methylation Scope: The study focuses on 5mC. Given that 6mA is also highly prevalent in H. pylori and often associated with Restriction-Modification (R-M) systems, a brief comment in the Discussion on why 6mA was not analyzed (or its potential role in future studies) would strengthen the epigenetic section.

Level of Interest

Please indicate how interesting you found the manuscript: Choose an item.

Quality of Written English

Please indicate the quality of language in the manuscript: Choose an item.

Declaration of Competing Interests

Please complete a declaration of competing interests, considering the following questions:

- Have you in the past five years received reimbursements, fees, funding, or salary from an organisation that may in any way gain or lose financially from the publication of this manuscript, either now or in the future?
- Do you hold any stocks or shares in an organisation that may in any way gain or lose financially from the publication of this manuscript, either now or in the future?
- Do you hold or are you currently applying for any patents relating to the content of the manuscript?
- Have you received reimbursements, fees, funding, or salary from an organization that holds or has applied for patents relating to the content of the manuscript?
- Do you have any other financial competing interests?
- Do you have any non-financial competing interests in relation to this paper?

If you can answer no to all of the above, write 'I declare that I have no competing interests' below. If your reply is yes to any, please give details below.

None.

I agree to the open peer review policy of the journal. I understand that my name will be included on my report to the authors and, if the manuscript is accepted for publication, my named report including any attachments I upload will be posted on the website along with the authors' responses. I agree for my report to be made available under an Open Access Creative Commons CC-BY license (http://creativecommons.org/licenses/by/4.0/). I understand that any comments which I do not wish to be included in my named report can be included as confidential comments to the editors, which will not be published.

I agree to the open peer review policy of the journal

To further support our reviewers, we have joined with Publons, where you can gain additional credit to further highlight your hard work (see: https://publons.com/journal/530/gigascience). On publication of this paper, your review will be automatically added to Publons, you can then choose whether or not to claim your Publons credit. I understand this statement.

Yes Choose an item.
