# Supplementary material for: Within-host diversity and phased variant analysis reveal structures and recombination of Helicobacter pylori subpopulations in stomach
Source: Gigascience. 2026 Apr 16;15:giag046. doi: 10.1093/gigascience/giag046 (PMC13188224; doi:10.1093/gigascience/giag046)
Supplement: giag046_Reviewer_2_Report_original_submission — Reviewer 2 -- 3/19/2026 [file giag046_reviewer_2_report_original_submission.pdf]

Reviewer Report

Title: Within-host diversity and phased variant analysis reveal structures and recombination of Helicobacter pylori subpopulations in stomach

Version: Original Submission **Date:** 3/19/2026

Reviewer name: Reviewer 2

Reviewer Comments to Author:

Zhang et al. collected multiple gastric biopsy samples colonized by H. pylori, evaluated the within-host genomic diversity of H. pylori, and uncovered the subpopulation structures in the human stomach. The findings are overall interesting, and these samples from the individuals provided, for the first time, a picture of how the sub-colonies are distributed in the stomach, including their relative abundance and genetic relationships, and particularly how resistance mutations emerged across subpopulations. The manuscript is generally well organized, and the figures are clear and easy to follow; however, the current version still requires careful revision to improve consistency and clarity.Major comments1. The sharing of iSNVs is presented in a pie chart, which reveals the subpopulations of H. pylori across different gastric regions. This is one of the main findings of the study. However, the authors did not show the sharing of SNPs between or among different gastric regions. Since this is a conventional and widely used method, analysis based on SNPs should be included.2. There is apparent discrepancy between the resistance-associated mutations and the antimicrobial susceptibility results. For example, in P24-A2, the subpopulation carrying the resistance-associated gyrA mutation appears to be the major one, whereas the isolate is reported as susceptible to levofloxacin. An explanation of this inconsistency would strengthen the interpretation of the result.3. The authors should add discussion about the clinical significance of these findings for H. pylori treatment.Minor comments1.Table 1 needs clearer annotation and more consistent anatomical labeling. In lines 105-108, the legend explains only R and S, but the meaning of "-" is not defined. In addition, the description of A1/A2 in lines 106-107 does not appear to be fully consistent with the terminology used elsewhere in the manuscript.2.There are a few singular/plural agreement issues. Line 142: "H. pylori isolate from the heteroresistance group" and line 305: "coexistence of minor and major subpopulation" likely should be plural.3. Methodological descriptions could be slightly clearer. In lines 411-412, the statement "the origin of replication was adjusted according to GC skew" would benefit from a brief indication of the software or procedure used.4.The presentation of resistance genotype and phenotype could be made more explicit. For example, the correspondence between resistance-associated mutations, phased subpopulations, and antimicrobial susceptibility results is not always easy to follow directly from the figures and text. It would be helpful to make these relationships more explicit, especially in the cases discussed around lines 239-262.5.The terminology for iSNVs should be used more consistently in the Results and figure legends. For example, lines 137-138 refer to "intra-region single nucleotide variations (iSNVs)", whereas lines 536-537 use "intra-host single nucleotide variants (iSNVs)". These should be standardized.

Level of Interest

Please indicate how interesting you found the manuscript: Choose an item.

Quality of Written English

Please indicate the quality of language in the manuscript: Choose an item.

Declaration of Competing Interests

Please complete a declaration of competing interests, considering the following questions:

- Have you in the past five years received reimbursements, fees, funding, or salary from an organisation that may in any way gain or lose financially from the publication of this manuscript, either now or in the future?
- Do you hold any stocks or shares in an organisation that may in any way gain or lose financially from the publication of this manuscript, either now or in the future?
- Do you hold or are you currently applying for any patents relating to the content of the manuscript?
- Have you received reimbursements, fees, funding, or salary from an organization that holds or has applied for patents relating to the content of the manuscript?
- Do you have any other financial competing interests?
- Do you have any non-financial competing interests in relation to this paper?

If you can answer no to all of the above, write 'I declare that I have no competing interests' below. If your reply is yes to any, please give details below.

I declare that I have no competing interests.

I agree to the open peer review policy of the journal. I understand that my name will be included on my report to the authors and, if the manuscript is accepted for publication, my named report including any attachments I upload will be posted on the website along with the authors' responses. I agree for my report to be made available under an Open Access Creative Commons CC-BY license (http://creativecommons.org/licenses/by/4.0/). I understand that any comments which I do not wish to be included in my named report can be included as confidential comments to the editors, which will not be published.

I agree to the open peer review policy of the journal

To further support our reviewers, we have joined with Publons, where you can gain additional credit to further highlight your hard work (see: https://publons.com/journal/530/gigascience). On publication of this paper, your review will be automatically added to Publons, you can then choose whether or not to claim your Publons credit. I understand this statement.

Yes Choose an item.
